# Correction: Assessment of temporomandibular disorders and their relationship with life quality and salivary biomarkers in patients with dentofacial deformities: A clinical observational study

**DOI:** 10.1371/journal.pone.0347249

**Published:** 2026-04-14

**Authors:** The PLOS One Staff

Fig 4 was uploaded incorrectly. Please see the correct Fig 4 here.

The publisher apologizes for the error.

**Fig 4:**
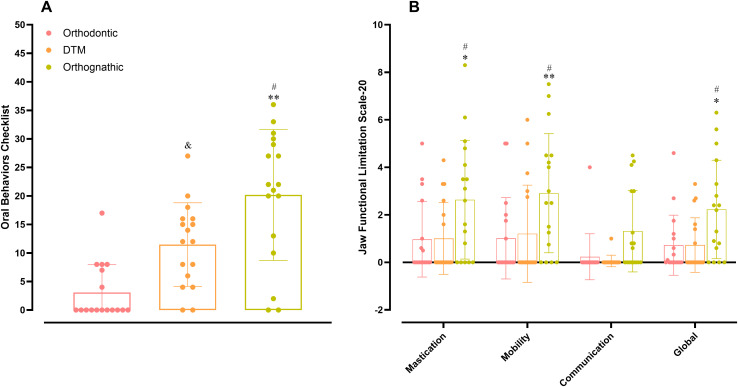
Analysis of jaw functional habits and limitations. (A) Comparison of oral behaviors checklist (OBC) assessment among orthodontic, TMD, and DFD participants. **p < 0.01 DFD vs. orthodontic; #*p* < 0.05 DFD vs. TMD; &*p* < 0.05. One-way ANOVA followed by Tukey’s multi-com*p*arison test. DFD, dentofacial deformity; TMD, temporomandibular disorder. (B) Analysis of orthodontic, TMD, and DFD groups in the different subscales of the jaw functional limitation scale-20. *p < 0.*05;* **p < 0.01 DFD vs. orthodontic; #*p* < 0.05 DFD vs. TMD. Two-way ANOVA followed by Tukey’s multi-com*p*arison test. Each column represents the mean, and the lines show the standard deviation (SD). The individual values are depicted as dot plots. DFD, dentofacial deformity; TMD, temporomandibular disorder.

## References

[1] Crescente BB, Bisatto NV, Rübensam G, Fritscher GG, Campos MM. Assessment of temporomandibular disorders and their relationship with life quality and salivary biomarkers in patients with dentofacial deformities: A clinical observational study. PLoS One. 2023;18(7):e0288914. doi: 10.1371/journal.pone.0288914 37471347 PMC10358945

