# Supplementary material for: Assessment of temporomandibular disorders and their relationship with life quality and salivary biomarkers in patients with dentofacial deformities: A clinical observational study
Source: PLoS One. 2023 Jul 20;18(7):e0288914. doi: 10.1371/journal.pone.0288914 (PMC10358945; doi:10.1371/journal.pone.0288914)
Supplement: S1 Fig — Frequencies (%) for PHQ-9 (A), GAD-7 (B), PHQ-15 (C), Oral behavior checklist (D), and chronic pain grade (E), as evaluated by DC-TMD Axis II for the orthodontic, TMD (temporomandibular disorder), and DFD (dentofacial deformity) groups. (PDF) [file pone.0288914.s001.pdf]

Supporting information

S1 Fig

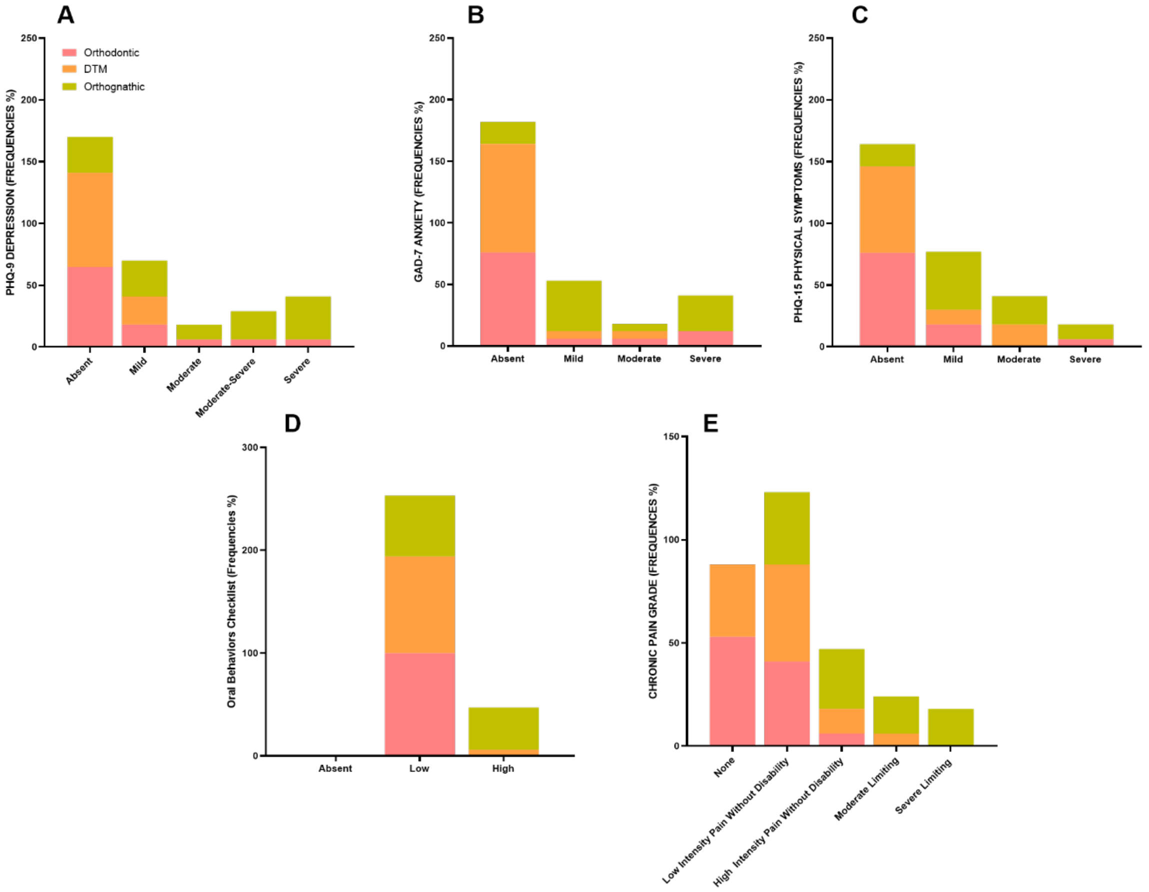

S1 Fig. Frequencies of Different Parameters of Axis II DC/TMD. Frequencies (%) for PHQ-9 (A), GAD-7 (B), PHQ-15 (C), Oral behavior checklist (D), and chronic pain grade (E), as evaluated by DC-TMD Axis II for the orthodontic, TMD (temporomandibular disorder), and DFD (dentofacial deformity) groups.
