# Supplementary material for: Assessment of temporomandibular disorders and their relationship with life quality and salivary biomarkers in patients with dentofacial deformities: A clinical observational study
Source: PLoS One. 2023 Jul 20;18(7):e0288914. doi: 10.1371/journal.pone.0288914 (PMC10358945; doi:10.1371/journal.pone.0288914)
Supplement: S1 File — Descriptive data and statistical analysis regarding the results presented in the manuscript. (PDF) [file pone.0288914.s002.pdf]

	Ordinary one-way ANOVA<br>ANOVA results					
1	Table Analyzed	OHIP-14 Ttotals				
2	Data sets analyzed	A-C				
3						
4	**ANOVA summary**					
5	F	20.49				
6	P value	<0.0001				
7	P value summary	****				
8	Significant diff. among means (P < 0.05)?	Yes				
9	R square	0.4606				
10						
11	**Brown-Forsythe test**					
12	F (DFn, DFd)	1.707 (2, 48)				
13	P value	0.1923				
14	P value summary	ns				
15	Are SDs significantly different (P < 0.05)?	No				
16						
17	**Bartlett's test**					
18	Bartlett's statistic (corrected)	2.581				
19	P value	0.2751				
20	P value summary	ns				
21	Are SDs significantly different (P < 0.05)?	No				
22						
23	**ANOVA table**	**SS**	**DF**	**MS**	**F (DFn, DFd)**	**P value**
24	Treatment (between columns)	3001	2	1500	F (2, 48) = 20.49	P<0.0001
25	Residual (within columns)	3515	48	73.23		
26	Total	6516	50			
27						
28	**Data summary**					
29	Number of treatments (columns)	3				
30	Number of values (total)	51				

Ordinary one-way ANOVA
Multiple comparisons

1	Number of families	1							
2	Number of comparisons per family	3							
3	Alpha	0.05							
4									
5	**Tukey's multiple comparisons test**	**Mean Diff.**	**95.00% CI of diff.**	**Significant?**	**Summary**	**Adjusted P Value**			
6	Orthodontic vs. DTM	-3.235	-10.33 to 3.863	No	ns	0.5174	A-B		
7	Orthodontic vs. Orthognathic	-17.65	-24.75 to -10.55	Yes	****	<0.0001	A-C		
8	DTM vs. Orthognathic	-14.41	-21.51 to -7.313	Yes	****	<0.0001	B-C		
9									
10	**Test details**	**Mean 1**	**Mean 2**	**Mean Diff.**	**SE of diff.**	**n1**	**n2**	**q**	**DF**
11	Orthodontic vs. DTM	5.176	8.412	-3.235	2.935	17	17	1.559	48
12	Orthodontic vs. Orthognathic	5.176	22.82	-17.65	2.935	17	17	8.503	48
13	DTM vs. Orthognathic	8.412	22.82	-14.41	2.935	17	17	6.944	48

2way ANOVA
ANOVA results

1	Table Analyzed	OHIP-14 Dimensions				
2						
3	**Two-way ANOVA**	Ordinary				
4	Alpha	0.05				
5						
6	**Source of Variation**	**% of total variation**	**P value**	**P value summary**	**Significant?**	
7	Interaction	4.496	0.0169	*	Yes	
8	Row Factor	12.70	<0.0001	****	Yes	
9	Column Factor	22.68	<0.0001	****	Yes	
10						
11	**ANOVA table**	**SS**	**DF**	**MS**	**F (DFn, DFd)**	**P value**
12	Interaction	83.43	12	6.952	F (12, 336) = 2.093	P=0.0169
13	Row Factor	235.6	6	39.27	F (6, 336) = 11.83	P<0.0001
14	Column Factor	420.8	2	210.4	F (2, 336) = 63.36	P<0.0001
15	Residual	1116	336	3.321		

2way ANOVA
Multiple comparisons

1	Within each row, compare columns (simp								
2									
3	Number of families	7							
4	Number of comparisons per family	3							
5	Alpha	0.05							
6									
7	**Tukey's multiple comparisons test**	**Mean Diff.**	**95.00% CI of diff.**	**Significant?**	**Summary**	**Adjusted P Value**			
8									
9	Functional Limitation								
10	Orthodontic vs. DTM	0.7059	-0.7656 to 2.177	No	ns	0.4967			
11	Orthodontic vs. Orthognathic	-0.8235	-2.295 to 0.6480	No	ns	0.3864			
12	DTM vs. Orthognathic	-1.529	-3.001 to -0.05790	Yes	*	0.0395			
13									
14	Physical Pain								
15	Orthodontic vs. DTM	-0.7647	-2.236 to 0.7068	No	ns	0.4402			
16	Orthodontic vs. Orthognathic	-1.765	-3.236 to -0.2932	Yes	*	0.0139			
17	DTM vs. Orthognathic	-1.000	-2.472 to 0.4715	No	ns	0.2471			
18									
19	Psychological Discomfort								
20	Orthodontic vs. DTM	-1.353	-2.824 to 0.1186	No	ns	0.0789			
21	Orthodontic vs. Orthognathic	-4.000	-5.472 to -2.528	Yes	****	<0.0001			
22	DTM vs. Orthognathic	-2.647	-4.119 to -1.176	Yes	****	<0.0001			
23									
24	Physical Disability								
25	Orthodontic vs. DTM	0.1765	-1.295 to 1.648	No	ns	0.9570			
26	Orthodontic vs. Orthognathic	-2.235	-3.707 to -0.7638	Yes	**	0.0012			
27	DTM vs. Orthognathic	-2.412	-3.883 to -0.9403	Yes	***	0.0004			
28									
29	Psychological Disability								
30	Orthodontic vs. DTM	-1.235	-2.707 to 0.2362	No	ns	0.1197			

	**2way ANOVA** Multiple comparisons								
31	Orthodontic vs. Orthognathic	-4.176	-5.648 to -2.705	Yes	****	<0.0001			
32	DTM vs. Orthognathic	-2.941	-4.413 to -1.470	Yes	****	<0.0001			
33									
34	Social Disability								
35	Orthodontic vs. DTM	-0.4118	-1.883 to 1.060	No	ns	0.7875			
36	Orthodontic vs. Orthognathic	-2.294	-3.766 to -0.8226	Yes	***	0.0008			
37	DTM vs. Orthognathic	-1.882	-3.354 to -0.4108	Yes	**	0.0079			
38									
39	Handicap								
40	Orthodontic vs. DTM	-0.1176	-1.589 to 1.354	No	ns	0.9807			
41	Orthodontic vs. Orthognathic	-2.118	-3.589 to -0.6461	Yes	**	0.0023			
42	DTM vs. Orthognathic	-2.000	-3.472 to -0.5285	Yes	**	0.0043			
43									
44									
45	**Test details**	**Mean 1**	**Mean 2**	**Mean Diff.**	**SE of diff.**	**N1**	**N2**	**q**	**DF**
46									
47	Functional Limitation								
48	Orthodontic vs. DTM	0.9412	0.2353	0.7059	0.6251	17	17	1.597	336.0
49	Orthodontic vs. Orthognathic	0.9412	1.765	-0.8235	0.6251	17	17	1.863	336.0
50	DTM vs. Orthognathic	0.2353	1.765	-1.529	0.6251	17	17	3.460	336.0
51									
52	Physical Pain								
53	Orthodontic vs. DTM	2.235	3.000	-0.7647	0.6251	17	17	1.730	336.0
54	Orthodontic vs. Orthognathic	2.235	4.000	-1.765	0.6251	17	17	3.993	336.0
55	DTM vs. Orthognathic	3.000	4.000	-1.000	0.6251	17	17	2.262	336.0
56									
57	Psychological Discomfort								
58	Orthodontic vs. DTM	0.9412	2.294	-1.353	0.6251	17	17	3.061	336.0
59	Orthodontic vs. Orthognathic	0.9412	4.941	-4.000	0.6251	17	17	9.050	336.0
60	DTM vs. Orthognathic	2.294	4.941	-2.647	0.6251	17	17	5.989	336.0

**2way ANOVA** Multiple comparisons									
61									
62	Physical Disability								
63	Orthodontic vs. DTM	0.6471	0.4706	0.1765	0.6251	17	17	0.3993	336.0
64	Orthodontic vs. Orthognathic	0.6471	2.882	-2.235	0.6251	17	17	5.057	336.0
65	DTM vs. Orthognathic	0.4706	2.882	-2.412	0.6251	17	17	5.457	336.0
66									
67	Psychological Disability								
68	Orthodontic vs. DTM	0.1765	1.412	-1.235	0.6251	17	17	2.795	336.0
69	Orthodontic vs. Orthognathic	0.1765	4.353	-4.176	0.6251	17	17	9.449	336.0
70	DTM vs. Orthognathic	1.412	4.353	-2.941	0.6251	17	17	6.654	336.0
71									
72	Social Disability								
73	Orthodontic vs. DTM	0.4118	0.8235	-0.4118	0.6251	17	17	0.9316	336.0
74	Orthodontic vs. Orthognathic	0.4118	2.706	-2.294	0.6251	17	17	5.190	336.0
75	DTM vs. Orthognathic	0.8235	2.706	-1.882	0.6251	17	17	4.259	336.0
76									
77	Handicap								
78	Orthodontic vs. DTM	0.05882	0.1765	-0.1176	0.6251	17	17	0.2662	336.0
79	Orthodontic vs. Orthognathic	0.05882	2.176	-2.118	0.6251	17	17	4.791	336.0
80	DTM vs. Orthognathic	0.1765	2.176	-2.000	0.6251	17	17	4.525	336.0

Ordinary one-way ANOVA<br>ANOVA results						
1	Table Analyzed	OBC				
2	Data sets analyzed	A-C				
3						
4	**ANOVA summary**					
5	F	17.84				
6	P value	<0.0001				
7	P value summary	****				
8	Significant diff. among means (P < 0.05)?	Yes				
9	R square	0.4263				
10						
11	**Brown-Forsythe test**					
12	F (DFn, DFd)	4.341 (2, 48)				
13	P value	0.0185				
14	P value summary	*				
15	Are SDs significantly different (P < 0.05)?	Yes				
16						
17	**Bartlett's test**					
18	Bartlett's statistic (corrected)	10.74				
19	P value	0.0047				
20	P value summary	**				
21	Are SDs significantly different (P < 0.05)?	Yes				
22						
23	**ANOVA table**	**SS**	**DF**	**MS**	**F (DFn, DFd)**	**P value**
24	Treatment (between columns)	2491	2	1245	F (2, 48) = 17.84	P<0.0001
25	Residual (within columns)	3352	48	69.83		
26	Total	5843	50			
27						
28	**Data summary**					
29	Number of treatments (columns)	3				
30	Number of values (total)	51				

	Ordinary one-way ANOVA<br>Multiple comparisons								
1	Number of families	1							
2	Number of comparisons per family	3							
3	Alpha	0.05							
4									
5	**Tukey's multiple comparisons test**	**Mean Diff.**	**95.00% CI of diff.**	**Significant?**	**Summary**	**Adjusted P Value**			
6	Orthodontic vs. DTM	-8.412	-15.34 to -1.480	Yes	*	0.0139	A-B		
7	Orthodontic vs. Orthognathic	-17.12	-24.05 to -10.19	Yes	****	<0.0001	A-C		
8	DTM vs. Orthognathic	-8.706	-15.64 to -1.774	Yes	*	0.0106	B-C		
9									
10	**Test details**	**Mean 1**	**Mean 2**	**Mean Diff.**	**SE of diff.**	**n1**	**n2**	**q**	**DF**
11	Orthodontic vs. DTM	3.059	11.47	-8.412	2.866	17	17	4.151	48
12	Orthodontic vs. Orthognathic	3.059	20.18	-17.12	2.866	17	17	8.446	48
13	DTM vs. Orthognathic	11.47	20.18	-8.706	2.866	17	17	4.296	48

Ordinary one-way ANOVA<br>ANOVA results						
1	Table Analyzed	OHIP-14 Ttotals				
2	Data sets analyzed	A-C				
3						
4	**ANOVA summary**					
5	F	20.49				
6	P value	<0.0001				
7	P value summary	****				
8	Significant diff. among means (P < 0.05)?	Yes				
9	R square	0.4606				
10						
11	**Brown-Forsythe test**					
12	F (DFn, DFd)	1.707 (2, 48)				
13	P value	0.1923				
14	P value summary	ns				
15	Are SDs significantly different (P < 0.05)?	No				
16						
17	**Bartlett's test**					
18	Bartlett's statistic (corrected)	2.581				
19	P value	0.2751				
20	P value summary	ns				
21	Are SDs significantly different (P < 0.05)?	No				
22						
23	**ANOVA table**	**SS**	**DF**	**MS**	**F (DFn, DFd)**	**P value**
24	Treatment (between columns)	3001	2	1500	F (2, 48) = 20.49	P<0.0001
25	Residual (within columns)	3515	48	73.23		
26	Total	6516	50			
27						
28	**Data summary**					
29	Number of treatments (columns)	3				
30	Number of values (total)	51				

Ordinary one-way ANOVA<br>Multiple comparisons									
1	Number of families	1							
2	Number of comparisons per family	3							
3	Alpha	0.05							
4									
5	**Tukey's multiple comparisons test**	**Mean Diff.**	**95.00% CI of diff.**	**Significant?**	**Summary**	**Adjusted P Value**			
6	Orthodontic vs. DTM	-3.235	-10.33 to 3.863	No	ns	0.5174	A-B		
7	Orthodontic vs. Orthognathic	-17.65	-24.75 to -10.55	Yes	****	<0.0001	A-C		
8	DTM vs. Orthognathic	-14.41	-21.51 to -7.313	Yes	****	<0.0001	B-C		
9									
10	**Test details**	**Mean 1**	**Mean 2**	**Mean Diff.**	**SE of diff.**	**n1**	**n2**	**q**	**DF**
11	Orthodontic vs. DTM	5.176	8.412	-3.235	2.935	17	17	1.559	48
12	Orthodontic vs. Orthognathic	5.176	22.82	-17.65	2.935	17	17	8.503	48
13	DTM vs. Orthognathic	8.412	22.82	-14.41	2.935	17	17	6.944	48

2way ANOVA
ANOVA results

1	Table Analyzed	OHIP-14 Dimensions				
2						
3	**Two-way ANOVA**	Ordinary				
4	Alpha	0.05				
5						
6	**Source of Variation**	**% of total variation**	**P value**	**P value summary**	**Significant?**	
7	Interaction	4.496	0.0169	*	Yes	
8	Row Factor	12.70	<0.0001	****	Yes	
9	Column Factor	22.68	<0.0001	****	Yes	
10						
11	**ANOVA table**	**SS**	**DF**	**MS**	**F (DFn, DFd)**	**P value**
12	Interaction	83.43	12	6.952	F (12, 336) = 2.093	P=0.0169
13	Row Factor	235.6	6	39.27	F (6, 336) = 11.83	P<0.0001
14	Column Factor	420.8	2	210.4	F (2, 336) = 63.36	P<0.0001
15	Residual	1116	336	3.321		

2way ANOVA<br>Multiple comparisons							
1	Within each row, compare columns (simple effects within rows)						
2							
3	Number of families	7					
4	Number of comparisons per family	3					
5	Alpha	0.05					
6							
7	**Tukey's multiple comparisons test**	**Mean Diff.**	**95.00% CI of diff.**	**Significant?**	**Summary**	**Adjusted P Value**	
8							
9	Functional Limitation						
10	Orthodontic vs. DTM	0.7059	-0.7656 to 2.177	No	ns	0.4967	
11	Orthodontic vs. Orthognathic	-0.8235	-2.295 to 0.6480	No	ns	0.3864	
12	DTM vs. Orthognathic	-1.529	-3.001 to -0.05790	Yes	*	0.0395	
13							
14	Physical Pain						
15	Orthodontic vs. DTM	-0.7647	-2.236 to 0.7068	No	ns	0.4402	
16	Orthodontic vs. Orthognathic	-1.765	-3.236 to -0.2932	Yes	*	0.0139	
17	DTM vs. Orthognathic	-1.000	-2.472 to 0.4715	No	ns	0.2471	
18							
19	Psychological Discomfort						
20	Orthodontic vs. DTM	-1.353	-2.824 to 0.1186	No	ns	0.0789	
21	Orthodontic vs. Orthognathic	-4.000	-5.472 to -2.528	Yes	****	<0.0001	
22	DTM vs. Orthognathic	-2.647	-4.119 to -1.176	Yes	****	<0.0001	
23							
24	Physical Disability						
25	Orthodontic vs. DTM	0.1765	-1.295 to 1.648	No	ns	0.9570	
26	Orthodontic vs. Orthognathic	-2.235	-3.707 to -0.7638	Yes	**	0.0012	
27	DTM vs. Orthognathic	-2.412	-3.883 to -0.9403	Yes	***	0.0004	
28							
29	Psychological Disability						
30	Orthodontic vs. DTM	-1.235	-2.707 to 0.2362	No	ns	0.1197	

	2way ANOVA<br>Multiple comparisons						
31	Orthodontic vs. Orthognathic	-4.176	-5.648 to -2.705	Yes	****	<0.0001	
32	DTM vs. Orthognathic	-2.941	-4.413 to -1.470	Yes	****	<0.0001	
33							
34	Social Disability						
35	Orthodontic vs. DTM	-0.4118	-1.883 to 1.060	No	ns	0.7875	
36	Orthodontic vs. Orthognathic	-2.294	-3.766 to -0.8226	Yes	***	0.0008	
37	DTM vs. Orthognathic	-1.882	-3.354 to -0.4108	Yes	**	0.0079	
38							
39	Handicap						
40	Orthodontic vs. DTM	-0.1176	-1.589 to 1.354	No	ns	0.9807	
41	Orthodontic vs. Orthognathic	-2.118	-3.589 to -0.6461	Yes	**	0.0023	
42	DTM vs. Orthognathic	-2.000	-3.472 to -0.5285	Yes	**	0.0043	
43							
44							
45	**Test details**	**Mean 1**	**Mean 2**	**Mean Diff.**	**SE of diff.**	**N1**	**N2**
46							
47	Functional Limitation						
48	Orthodontic vs. DTM	0.9412	0.2353	0.7059	0.6251	17	17
49	Orthodontic vs. Orthognathic	0.9412	1.765	-0.8235	0.6251	17	17
50	DTM vs. Orthognathic	0.2353	1.765	-1.529	0.6251	17	17
51							
52	Physical Pain						
53	Orthodontic vs. DTM	2.235	3.000	-0.7647	0.6251	17	17
54	Orthodontic vs. Orthognathic	2.235	4.000	-1.765	0.6251	17	17
55	DTM vs. Orthognathic	3.000	4.000	-1.000	0.6251	17	17
56							
57	Psychological Discomfort						
58	Orthodontic vs. DTM	0.9412	2.294	-1.353	0.6251	17	17
59	Orthodontic vs. Orthognathic	0.9412	4.941	-4.000	0.6251	17	17
60	DTM vs. Orthognathic	2.294	4.941	-2.647	0.6251	17	17

**2way ANOVA**<br>Multiple comparisons							
61							
62	Physical Disability						
63	Orthodontic vs. DTM	0.6471	0.4706	0.1765	0.6251	17	17
64	Orthodontic vs. Orthognathic	0.6471	2.882	-2.235	0.6251	17	17
65	DTM vs. Orthognathic	0.4706	2.882	-2.412	0.6251	17	17
66							
67	Psychological Disability						
68	Orthodontic vs. DTM	0.1765	1.412	-1.235	0.6251	17	17
69	Orthodontic vs. Orthognathic	0.1765	4.353	-4.176	0.6251	17	17
70	DTM vs. Orthognathic	1.412	4.353	-2.941	0.6251	17	17
71							
72	Social Disability						
73	Orthodontic vs. DTM	0.4118	0.8235	-0.4118	0.6251	17	17
74	Orthodontic vs. Orthognathic	0.4118	2.706	-2.294	0.6251	17	17
75	DTM vs. Orthognathic	0.8235	2.706	-1.882	0.6251	17	17
76							
77	Handicap						
78	Orthodontic vs. DTM	0.05882	0.1765	-0.1176	0.6251	17	17
79	Orthodontic vs. Orthognathic	0.05882	2.176	-2.118	0.6251	17	17
80	DTM vs. Orthognathic	0.1765	2.176	-2.000	0.6251	17	17

1		
2		
3		
4		
5		
6		
7		
8		
9		
10		
11		
12		
13		
14		
15		
16		
17		
18		
19		
20		
21		
22		
23		
24		
25		
26		
27		
28		
29		
30		

31		
32		
33		
34		
35		
36		
37		
38		
39		
40		
41		
42		
43		
44		
45	**q**	**DF**
46		
47		
48	1.597	336.0
49	1.863	336.0
50	3.460	336.0
51		
52		
53	1.730	336.0
54	3.993	336.0
55	2.262	336.0
56		
57		
58	3.061	336.0
59	9.050	336.0
60	5.989	336.0

61		
62		
63	0.3993	336.0
64	5.057	336.0
65	5.457	336.0
66		
67		
68	2.795	336.0
69	9.449	336.0
70	6.654	336.0
71		
72		
73	0.9316	336.0
74	5.190	336.0
75	4.259	336.0
76		
77		
78	0.2662	336.0
79	4.791	336.0
80	4.525	336.0

	Ordinary one-way ANOVA<br>ANOVA results					
1	Table Analyzed	OHIP-14 Ttotals				
2	Data sets analyzed	A-C				
3						
4	**ANOVA summary**					
5	F	20.49				
6	P value	<0.0001				
7	P value summary	****				
8	Significant diff. among means (P < 0.05)?	Yes				
9	R square	0.4606				
10						
11	**Brown-Forsythe test**					
12	F (DFn, DFd)	1.707 (2, 48)				
13	P value	0.1923				
14	P value summary	ns				
15	Are SDs significantly different (P < 0.05)?	No				
16						
17	**Bartlett's test**					
18	Bartlett's statistic (corrected)	2.581				
19	P value	0.2751				
20	P value summary	ns				
21	Are SDs significantly different (P < 0.05)?	No				
22						
23	**ANOVA table**	**SS**	**DF**	**MS**	**F (DFn, DFd)**	**P value**
24	Treatment (between columns)	3001	2	1500	F (2, 48) = 20.4	P<0.0001
25	Residual (within columns)	3515	48	73.23		
26	Total	6516	50			
27						
28	**Data summary**					
29	Number of treatments (columns)	3				
30	Number of values (total)	51				

	Ordinary one-way ANOVA<br>Multiple comparisons								
1	Number of families	1							
2	Number of comparisons per family	3							
3	Alpha	0.05							
4									
5	**Tukey's multiple comparisons test**	**Mean Diff.**	**95.00% CI of diff.**	**Significant?**	**Summary**	**Adjusted P Value**			
6	Orthodontic vs. DTM	-3.235	-10.33 to 3.863	No	ns	0.5174	A-B		
7	Orthodontic vs. Orthognathic	-17.65	-24.75 to -10.55	Yes	****	<0.0001	A-C		
8	DTM vs. Orthognathic	-14.41	-21.51 to -7.313	Yes	****	<0.0001	B-C		
9									
10	**Test details**	**Mean 1**	**Mean 2**	**Mean Diff.**	**SE of diff.**	**n1**	**n2**	**q**	**DF**
11	Orthodontic vs. DTM	5.176	8.412	-3.235	2.935	17	17	1.559	48
12	Orthodontic vs. Orthognathic	5.176	22.82	-17.65	2.935	17	17	8.503	48
13	DTM vs. Orthognathic	8.412	22.82	-14.41	2.935	17	17	6.944	48

2way ANOVA
ANOVA results

1	Table Analyzed	OHIP-14 Dimensions				
2						
3	**Two-way ANOVA**	Ordinary				
4	Alpha	0.05				
5						
6	**Source of Variation**	**% of total variation**	**P value**	**P value summary**	**Significant?**	
7	Interaction	4.496	0.0169	*	Yes	
8	Row Factor	12.70	<0.0001	****	Yes	
9	Column Factor	22.68	<0.0001	****	Yes	
10						
11	**ANOVA table**	**SS**	**DF**	**MS**	**F (DFn, DFd)**	**P value**
12	Interaction	83.43	12	6.952	F (12, 336) = 2.093	P=0.0169
13	Row Factor	235.6	6	39.27	F (6, 336) = 11.83	P<0.0001
14	Column Factor	420.8	2	210.4	F (2, 336) = 63.36	P<0.0001
15	Residual	1116	336	3.321		

2way ANOVA
Multiple comparisons

1	Within each row, compare columns (s					
2						
3	Number of families	7				
4	Number of comparisons per family	3				
5	Alpha	0.05				
6						
7	**Tukey's multiple comparisons tes**	**Mean Diff.**	**95.00% CI of diff.**	**Significant?**	**Summary**	**Adjusted P Value**
8						
9	Functional Limitation					
10	Orthodontic vs. DTM	0.7059	-0.7656 to 2.177	No	ns	0.4967
11	Orthodontic vs. Orthognathic	-0.8235	-2.295 to 0.6480	No	ns	0.3864
12	DTM vs. Orthognathic	-1.529	-3.001 to -0.05790	Yes	*	0.0395
13						
14	Physical Pain					
15	Orthodontic vs. DTM	-0.7647	-2.236 to 0.7068	No	ns	0.4402
16	Orthodontic vs. Orthognathic	-1.765	-3.236 to -0.2932	Yes	*	0.0139
17	DTM vs. Orthognathic	-1.000	-2.472 to 0.4715	No	ns	0.2471
18						
19	Psychological Discomfort					
20	Orthodontic vs. DTM	-1.353	-2.824 to 0.1186	No	ns	0.0789
21	Orthodontic vs. Orthognathic	-4.000	-5.472 to -2.528	Yes	****	<0.0001
22	DTM vs. Orthognathic	-2.647	-4.119 to -1.176	Yes	****	<0.0001
23						
24	Physical Disability					
25	Orthodontic vs. DTM	0.1765	-1.295 to 1.648	No	ns	0.9570
26	Orthodontic vs. Orthognathic	-2.235	-3.707 to -0.7638	Yes	**	0.0012
27	DTM vs. Orthognathic	-2.412	-3.883 to -0.9403	Yes	***	0.0004
28						
29	Psychological Disability					
30	Orthodontic vs. DTM	-1.235	-2.707 to 0.2362	No	ns	0.1197

**2way ANOVA** Multiple comparisons									
31	Orthodontic vs. Orthognathic	-4.176	-5.648 to -2.705	Yes	****	<0.0001			
32	DTM vs. Orthognathic	-2.941	-4.413 to -1.470	Yes	****	<0.0001			
33									
34	Social Disability								
35	Orthodontic vs. DTM	-0.4118	-1.883 to 1.060	No	ns	0.7875			
36	Orthodontic vs. Orthognathic	-2.294	-3.766 to -0.8226	Yes	***	0.0008			
37	DTM vs. Orthognathic	-1.882	-3.354 to -0.4108	Yes	**	0.0079			
38									
39	Handicap								
40	Orthodontic vs. DTM	-0.1176	-1.589 to 1.354	No	ns	0.9807			
41	Orthodontic vs. Orthognathic	-2.118	-3.589 to -0.6461	Yes	**	0.0023			
42	DTM vs. Orthognathic	-2.000	-3.472 to -0.5285	Yes	**	0.0043			
43									
44									
45	**Test details**	**Mean 1**	**Mean 2**	**Mean Diff.**	**SE of diff.**	**N1**	**N2**	**q**	**DF**
46									
47	Functional Limitation								
48	Orthodontic vs. DTM	0.9412	0.2353	0.7059	0.6251	17	17	1.597	336.0
49	Orthodontic vs. Orthognathic	0.9412	1.765	-0.8235	0.6251	17	17	1.863	336.0
50	DTM vs. Orthognathic	0.2353	1.765	-1.529	0.6251	17	17	3.460	336.0
51									
52	Physical Pain								
53	Orthodontic vs. DTM	2.235	3.000	-0.7647	0.6251	17	17	1.730	336.0
54	Orthodontic vs. Orthognathic	2.235	4.000	-1.765	0.6251	17	17	3.993	336.0
55	DTM vs. Orthognathic	3.000	4.000	-1.000	0.6251	17	17	2.262	336.0
56									
57	Psychological Discomfort								
58	Orthodontic vs. DTM	0.9412	2.294	-1.353	0.6251	17	17	3.061	336.0
59	Orthodontic vs. Orthognathic	0.9412	4.941	-4.000	0.6251	17	17	9.050	336.0
60	DTM vs. Orthognathic	2.294	4.941	-2.647	0.6251	17	17	5.989	336.0

**2way ANOVA**<br>Multiple comparisons									
61									
62	Physical Disability								
63	Orthodontic vs. DTM	0.6471	0.4706	0.1765	0.6251	17	17	0.3993	336.0
64	Orthodontic vs. Orthognathic	0.6471	2.882	-2.235	0.6251	17	17	5.057	336.0
65	DTM vs. Orthognathic	0.4706	2.882	-2.412	0.6251	17	17	5.457	336.0
66									
67	Psychological Disability								
68	Orthodontic vs. DTM	0.1765	1.412	-1.235	0.6251	17	17	2.795	336.0
69	Orthodontic vs. Orthognathic	0.1765	4.353	-4.176	0.6251	17	17	9.449	336.0
70	DTM vs. Orthognathic	1.412	4.353	-2.941	0.6251	17	17	6.654	336.0
71									
72	Social Disability								
73	Orthodontic vs. DTM	0.4118	0.8235	-0.4118	0.6251	17	17	0.9316	336.0
74	Orthodontic vs. Orthognathic	0.4118	2.706	-2.294	0.6251	17	17	5.190	336.0
75	DTM vs. Orthognathic	0.8235	2.706	-1.882	0.6251	17	17	4.259	336.0
76									
77	Handicap								
78	Orthodontic vs. DTM	0.05882	0.1765	-0.1176	0.6251	17	17	0.2662	336.0
79	Orthodontic vs. Orthognathic	0.05882	2.176	-2.118	0.6251	17	17	4.791	336.0
80	DTM vs. Orthognathic	0.1765	2.176	-2.000	0.6251	17	17	4.525	336.0

**Ordinary one-way ANOVA**<br>ANOVA results						
1	Table Analyzed	PHQ9-DEPRESSIC				
2	Data sets analyzed	A-C				
3						
4	**ANOVA summary**					
5	F	9.019				
6	P value	0.0005				
7	P value summary	***				
8	Significant diff. among means (P < 0.05)?	Yes				
9	R square	0.2731				
10						
11	**Brown-Forsythe test**					
12	F (DFn, DFd)	2.372 (2, 48)				
13	P value	0.1041				
14	P value summary	ns				
15	Are SDs significantly different (P < 0.05)?	No				
16						
17	**Bartlett's test**					
18	Bartlett's statistic (corrected)	11.85				
19	P value	0.0027				
20	P value summary	**				
21	Are SDs significantly different (P < 0.05)?	Yes				
22						
23	**ANOVA table**	**SS**	**DF**	**MS**	**F (DFn, DFd)**	**P value**
24	Treatment (between columns)	580.9	2	290.4	F (2, 48) = 9.0	P=0.0005
25	Residual (within columns)	1546	48	32.20		
26	Total	2127	50			
27						
28	**Data summary**					
29	Number of treatments (columns)	3				
30	Number of values (total)	51				

Ordinary one-way ANOVA
Multiple comparisons

1	Number of families	1							
2	Number of comparisons per family	3							
3	Alpha	0.05							
4									
5	**Tukey's multiple comparisons test**	**Mean Diff.**	**95.00% CI of diff.**	**Significant?**	**Summary**	**Adjusted P Value**			
6	Orthodontic vs. DTM	2.706	-2.002 to 7.413	No	ns	0.3540	A-B		
7	Orthodontic vs. Orthognathic	-5.412	-10.12 to -0.7043	Yes	*	0.0207	A-C		
8	DTM vs. Orthognathic	-8.118	-12.83 to -3.410	Yes	***	0.0004	B-C		
9									
10	**Test details**	**Mean 1**	**Mean 2**	**Mean Diff.**	**SE of diff.**	**n1**	**n2**	**q**	**DF**
11	Orthodontic vs. DTM	4.647	1.941	2.706	1.946	17	17	1.966	48
12	Orthodontic vs. Orthognathic	4.647	10.06	-5.412	1.946	17	17	3.932	48
13	DTM vs. Orthognathic	1.941	10.06	-8.118	1.946	17	17	5.898	48

Ordinary one-way ANOVA<br>ANOVA results						
1	Table Analyzed	GAD-7-ANXIETY				
2	Data sets analyzed	A-C				
3						
4	**ANOVA summary**					
5	F	8.323				
6	P value	0.0008				
7	P value summary	***				
8	Significant diff. among means (P < 0.05)?	Yes				
9	R square	0.2575				
10						
11	**Brown-Forsythe test**					
12	F (DFn, DFd)	1.684 (2, 48)				
13	P value	0.1964				
14	P value summary	ns				
15	Are SDs significantly different (P < 0.05)?	No				
16						
17	**Bartlett's test**					
18	Bartlett's statistic (corrected)	10.15				
19	P value	0.0063				
20	P value summary	**				
21	Are SDs significantly different (P < 0.05)?	Yes				
22						
23	**ANOVA table**	**SS**	**DF**	**MS**	**F (DFn, DFd)**	**P value**
24	Treatment (between columns)	448.0	2	224.0	F (2, 48) = 8.3:	P=0.0008
25	Residual (within columns)	1292	48	26.92		
26	Total	1740	50			
27						
28	**Data summary**					
29	Number of treatments (columns)	3				
30	Number of values (total)	51				

Ordinary one-way ANOVA<br>Multiple comparisons									
1	Number of families	1							
2	Number of comparisons per family	3							
3	Alpha	0.05							
4									
5	**Tukey's multiple comparisons test**	**Mean Diff.**	**95.00% CI of diff.**	**Significant?**	**Summary**	**Adjusted P Value**			
6	Orthodontic vs. DTM	2.059	-2.245 to 6.363	No	ns	0.4844	A-B		
7	Orthodontic vs. Orthognathic	-5.000	-9.304 to -0.6963	Yes	*	0.0192	A-C		
8	DTM vs. Orthognathic	-7.059	-11.36 to -2.755	Yes	***	0.0007	B-C		
9									
10	**Test details**	**Mean 1**	**Mean 2**	**Mean Diff.**	**SE of diff.**	**n1**	**n2**	**q**	**DF**
11	Orthodontic vs. DTM	3.824	1.765	2.059	1.780	17	17	1.636	48
12	Orthodontic vs. Orthognathic	3.824	8.824	-5.000	1.780	17	17	3.974	48
13	DTM vs. Orthognathic	1.765	8.824	-7.059	1.780	17	17	5.610	48

Ordinary one-way ANOVA<br>ANOVA results						
1	Table Analyzed	PHQ-15-PHYSICAL SYMPTOM				
2	Data sets analyzed	A-C				
3						
4	**ANOVA summary**					
5	F	10.05				
6	P value	0.0002				
7	P value summary	***				
8	Significant diff. among means (P < 0.05)?	Yes				
9	R square	0.2951				
10						
11	**Brown-Forsythe test**					
12	F (DFn, DFd)	0.3273 (2, 48)				
13	P value	0.7224				
14	P value summary	ns				
15	Are SDs significantly different (P < 0.05)?	No				
16						
17	**Bartlett's test**					
18	Bartlett's statistic (corrected)	0.2125				
19	P value	0.8992				
20	P value summary	ns				
21	Are SDs significantly different (P < 0.05)?	No				
22						
23	**ANOVA table**	**SS**	**DF**	**MS**	**F (DFn, DFd)**	**P value**
24	Treatment (between columns)	300.0	2	150.0	F (2, 48) = 10.05	P=0.0002
25	Residual (within columns)	716.6	48	14.93		
26	Total	1017	50			
27						
28	**Data summary**					
29	Number of treatments (columns)	3				
30	Number of values (total)	51				

**Ordinary one-way ANOVA**<br>Multiple comparisons								
1	Number of families	1						
2	Number of comparisons per family	2						
3	Alpha	0.05						
4								
5	**Dunnett's multiple comparisons test**	**Mean Diff.**	**95.00% CI of diff.**	**Significant?**	**Summary**	**Adjusted P Value**	**A-?**	
6	Orthodontic vs. DTM	-0.8824	-3.903 to 2.138	No	ns	0.7312	B	DTM
7	Orthodontic vs. Orthognathic	-5.529	-8.550 to -2.509	Yes	***	0.0003	C	Orthognathic
8								
9	**Test details**	**Mean 1**	**Mean 2**	**Mean Diff.**	**SE of diff.**	**n1**	**n2**	**q**
10	Orthodontic vs. DTM	3.412	4.294	-0.8824	1.325	17	17	0.6658
11	Orthodontic vs. Orthognathic	3.412	8.941	-5.529	1.325	17	17	4.172

1	
2	
3	
4	
5	
6	
7	
8	
9	**DF**
10	48
11	48

**Ordinary one-way ANOVA**<br>ANOVA results						
1	Table Analyzed	PHQ-15-PHYSICAL SYMPTOM				
2	Data sets analyzed	A-C				
3						
4	**ANOVA summary**					
5	F	10.05				
6	P value	0.0002				
7	P value summary	***				
8	Significant diff. among means (P < 0.05)?	Yes				
9	R square	0.2951				
10						
11	**Brown-Forsythe test**					
12	F (DFn, DFd)	0.3273 (2, 48)				
13	P value	0.7224				
14	P value summary	ns				
15	Are SDs significantly different (P < 0.05)?	No				
16						
17	**Bartlett's test**					
18	Bartlett's statistic (corrected)	0.2125				
19	P value	0.8992				
20	P value summary	ns				
21	Are SDs significantly different (P < 0.05)?	No				
22						
23	**ANOVA table**	**SS**	**DF**	**MS**	**F (DFn, DFd)**	**P value**
24	Treatment (between columns)	300.0	2	150.0	F (2, 48) = 10.05	P=0.0002
25	Residual (within columns)	716.6	48	14.93		
26	Total	1017	50			
27						
28	**Data summary**					
29	Number of treatments (columns)	3				
30	Number of values (total)	51				

Ordinary one-way ANOVA<br>Multiple comparisons									
1	Number of families	1							
2	Number of comparisons per family	3							
3	Alpha	0.05							
4									
5	**Tukey's multiple comparisons test**	**Mean Diff.**	**95.00% CI of diff.**	**Significant?**	**Summary**	**Adjusted P Value**			
6	Orthodontic vs. DTM	-0.8824	-4.088 to 2.323	No	ns	0.7843	A-B		
7	Orthodontic vs. Orthognathic	-5.529	-8.735 to -2.324	Yes	***	0.0004	A-C		
8	DTM vs. Orthognathic	-4.647	-7.852 to -1.442	Yes	**	0.0028	B-C		
9									
10	**Test details**	**Mean 1**	**Mean 2**	**Mean Diff.**	**SE of diff.**	**n1**	**n2**	**q**	**DF**
11	Orthodontic vs. DTM	3.412	4.294	-0.8824	1.325	17	17	0.9416	48
12	Orthodontic vs. Orthognathic	3.412	8.941	-5.529	1.325	17	17	5.901	48
13	DTM vs. Orthognathic	4.294	8.941	-4.647	1.325	17	17	4.959	48

**Ordinary one-way ANOVA** ANOVA results						
1	Table Analyzed	OBC				
2	Data sets analyzed	A-C				
3						
4	**ANOVA summary**					
5	F	17.84				
6	P value	<0.0001				
7	P value summary	****				
8	Significant diff. among means (P < 0.05)?	Yes				
9	R square	0.4263				
10						
11	**Brown-Forsythe test**					
12	F (DFn, DFd)	4.341 (2, 48)				
13	P value	0.0185				
14	P value summary	*				
15	Are SDs significantly different (P < 0.05)?	Yes				
16						
17	**Bartlett's test**					
18	Bartlett's statistic (corrected)	10.74				
19	P value	0.0047				
20	P value summary	**				
21	Are SDs significantly different (P < 0.05)?	Yes				
22						
23	**ANOVA table**	**SS**	**DF**	**MS**	**F (DFn, DFd)**	**P value**
24	Treatment (between columns)	2491	2	1245	F (2, 48) = 17.84	P<0.0001
25	Residual (within columns)	3352	48	69.83		
26	Total	5843	50			
27						
28	**Data summary**					
29	Number of treatments (columns)	3				
30	Number of values (total)	51				

**Ordinary one-way ANOVA**<br>Multiple comparisons									
1	Number of families	1							
2	Number of comparisons per family	3							
3	Alpha	0.05							
4									
5	**Tukey's multiple comparisons test**	**Mean Diff.**	**95.00% CI of diff.**	**Significant?**	**Summary**	**Adjusted P Value**			
6	Orthodontic vs. DTM	-8.412	-15.34 to -1.480	Yes	*	0.0139	A-B		
7	Orthodontic vs. Orthognathic	-17.12	-24.05 to -10.19	Yes	****	<0.0001	A-C		
8	DTM vs. Orthognathic	-8.706	-15.64 to -1.774	Yes	*	0.0106	B-C		
9									
10	**Test details**	**Mean 1**	**Mean 2**	**Mean Diff.**	**SE of diff.**	**n1**	**n2**	**q**	**DF**
11	Orthodontic vs. DTM	3.059	11.47	-8.412	2.866	17	17	4.151	48
12	Orthodontic vs. Orthognathic	3.059	20.18	-17.12	2.866	17	17	8.446	48
13	DTM vs. Orthognathic	11.47	20.18	-8.706	2.866	17	17	4.296	48

**Kruskal-Wallis test**<br>ANOVA results		
1	Table Analyzed	OBC
2		
3	**Kruskal-Wallis test**	
4	P value	<0.0001
5	Exact or approximate P value?	Approximate
6	P value summary	****
7	Do the medians vary signif. (P < 0.05)?	Yes
8	Number of groups	3
9	Kruskal-Wallis statistic	21.31
10		
11	**Data summary**	
12	Number of treatments (columns)	3
13	Number of values (total)	51

Kruskal-Wallis test<br>Multiple comparisons							
1	Number of families	1					
2	Number of comparisons per family	3					
3	Alpha	0.05					
4							
5	**Dunn's multiple comparisons test**	**Mean rank diff.**	**Significant?**	**Summary**	**Adjusted P Value**		
6	Orthodontic vs. DTM	-13.41	Yes	*	0.0230	A-B	
7	Orthodontic vs. Orthognathic	-23.12	Yes	****	<0.0001	A-C	
8	DTM vs. Orthognathic	-9.706	No	ns	0.1609	B-C	
9							
10	**Test details**	**Mean rank 1**	**Mean rank 2**	**Mean rank diff.**	**n1**	**n2**	
11	Orthodontic vs. DTM	13.82	27.24	-13.41	17	17	
12	Orthodontic vs. Orthognathic	13.82	36.94	-23.12	17	17	
13	DTM vs. Orthognathic	27.24	36.94	-9.706	17	17	

Ordinary one-way ANOVA<br>ANOVA results						
1	Table Analyzed	GCPS CPI				
2	Data sets analyzed	A-C				
3						
4	**ANOVA summary**					
5	F	8.562				
6	P value	0.0007				
7	P value summary	***				
8	Significant diff. among means (P < 0.05)?	Yes				
9	R square	0.2629				
10						
11	**Brown-Forsythe test**					
12	F (DFn, DFd)	0.08210 (2, 48)				
13	P value	0.9213				
14	P value summary	ns				
15	Are SDs significantly different (P < 0.05)?	No				
16						
17	**Bartlett's test**					
18	Bartlett's statistic (corrected)	0.02380				
19	P value	0.9882				
20	P value summary	ns				
21	Are SDs significantly different (P < 0.05)?	No				
22						
23	**ANOVA table**	**SS**	**DF**	**MS**	**F (DFn, DFd)**	**P value**
24	Treatment (between columns)	9487	2	4744	F (2, 48) = 8.5	P=0.0007
25	Residual (within columns)	26593	48	554.0		
26	Total	36080	50			
27						
28	**Data summary**					
29	Number of treatments (columns)	3				
30	Number of values (total)	51				

**Ordinary one-way ANOVA**<br>Multiple comparisons									
1	Number of families	1							
2	Number of comparisons per family	3							
3	Alpha	0.05							
4									
5	**Tukey's multiple comparisons test**	**Mean Diff.**	**95.00% CI of diff.**	**Significant?**	**Summary**	**Adjusted P Value**			
6	Orthodontic vs. DTM	-5.353	-24.88 to 14.17	No	ns	0.7859	A-B		
7	Orthodontic vs. Orthognathic	-31.24	-50.76 to -11.71	Yes	***	0.0009	A-C		
8	DTM vs. Orthognathic	-25.88	-45.41 to -6.357	Yes	**	0.0067	B-C		
9									
10	**Test details**	**Mean 1**	**Mean 2**	**Mean Diff.**	**SE of diff.**	**n1**	**n2**	**q**	**DF**
11	Orthodontic vs. DTM	20.00	25.35	-5.353	8.073	17	17	0.9377	48
12	Orthodontic vs. Orthognathic	20.00	51.24	-31.24	8.073	17	17	5.472	48
13	DTM vs. Orthognathic	25.35	51.24	-25.88	8.073	17	17	4.534	48

Ordinary one-way ANOVA<br>ANOVA results						
1	Table Analyzed	CPG				
2	Data sets analyzed	A-C				
3						
4	**ANOVA summary**					
5	F	15.95				
6	P value	<0.0001				
7	P value summary	****				
8	Significant diff. among means (P < 0.05)?	Yes				
9	R square	0.3993				
10						
11	**Brown-Forsythe test**					
12	F (DFn, DFd)	1.450 (2, 48)				
13	P value	0.2446				
14	P value summary	ns				
15	Are SDs significantly different (P < 0.05)?	No				
16						
17	**Bartlett's test**					
18	Bartlett's statistic (corrected)	5.286				
19	P value	0.0712				
20	P value summary	ns				
21	Are SDs significantly different (P < 0.05)?	No				
22						
23	**ANOVA table**	**SS**	**DF**	**MS**	**F (DFn, DFd)**	**P value**
24	Treatment (between columns)	25.57	2	12.78	F (2, 48) = 15.95	P<0.0001
25	Residual (within columns)	38.47	48	0.8015		
26	Total	64.04	50			
27						
28	**Data summary**					
29	Number of treatments (columns)	3				
30	Number of values (total)	51				

Ordinary one-way ANOVA<br>Multiple comparisons									
1	Number of families	1							
2	Number of comparisons per family	3							
3	Alpha	0.05							
4									
5	**Tukey's multiple comparisons test**	**Mean Diff.**	**95.00% CI of diff.**	**Significant?**	**Summary**	**Adjusted P Value**			
6	Orthodontic vs. DTM	-0.3529	-1.096 to 0.3897	No	ns	0.4889	A-B		
7	Orthodontic vs. Orthognathic	-1.647	-2.390 to -0.9044	Yes	****	<0.0001	A-C		
8	DTM vs. Orthognathic	-1.294	-2.037 to -0.5515	Yes	***	0.0003	B-C		
9									
10	**Test details**	**Mean 1**	**Mean 2**	**Mean Diff.**	**SE of diff.**	**n1**	**n2**	**q**	**DF**
11	Orthodontic vs. DTM	0.5294	0.8824	-0.3529	0.3071	17	17	1.625	48
12	Orthodontic vs. Orthognathic	0.5294	2.176	-1.647	0.3071	17	17	7.586	48
13	DTM vs. Orthognathic	0.8824	2.176	-1.294	0.3071	17	17	5.960	48

2way ANOVA<br>ANOVA results						
1	Table Analyzed	JFLS-20 Dimensions				
2						
3	**Two-way ANOVA**	Ordinary				
4	Alpha	0.05				
5						
6	**Source of Variation**	**% of total variation**	**P value**	**P value summary**	**Significant?**	
7	Interaction	0.4272	0.9839	ns	No	
8	Row Factor	5.711	0.0038	**	Yes	
9	Column Factor	14.73	<0.0001	****	Yes	
10						
11	**ANOVA table**	**SS**	**DF**	**MS**	**F (DFn, DFd)**	**P value**
12	Interaction	3.072	6	0.5120	F (6, 192) = 0.1728	P=0.9839
13	Row Factor	41.07	3	13.69	F (3, 192) = 4.619	P=0.0038
14	Column Factor	105.9	2	52.96	F (2, 192) = 17.87	P<0.0001
15	Residual	569.1	192	2.964		

2way ANOVA
Multiple comparisons

1	Within each row, compare columns (s								
2									
3	Number of families	4							
4	Number of comparisons per family	3							
5	Alpha	0.05							
6									
7	**Tukey's multiple comparisons tes**	**Mean Diff.**	**95.00% CI of diff.**	**Significant?**	**Summary**	**Adjusted P Value**			
8									
9	Mastication								
10	Orthodontic vs. DTM	-0.03529	-1.430 to 1.359	No	ns	0.9980			
11	Orthodontic vs. Orthognathic	-1.665	-3.059 to -0.2699	Yes	*	0.0146			
12	DTM vs. Orthognathic	-1.629	-3.024 to -0.2346	Yes	*	0.0174			
13									
14	Mobility								
15	Orthodontic vs. DTM	-0.1912	-1.586 to 1.204	No	ns	0.9439			
16	Orthodontic vs. Orthognathic	-1.894	-3.289 to -0.4993	Yes	**	0.0045			
17	DTM vs. Orthognathic	-1.703	-3.098 to -0.3082	Yes	*	0.0121			
18									
19	Communication								
20	Orthodontic vs. DTM	0.1765	-1.218 to 1.571	No	ns	0.9520			
21	Orthodontic vs. Orthognathic	-1.076	-2.471 to 0.3183	No	ns	0.1648			
22	DTM vs. Orthognathic	-1.253	-2.648 to 0.1418	No	ns	0.0881			
23									
24	Global								
25	Orthodontic vs. DTM	-0.007059	-1.402 to 1.388	No	ns	>0.9999			
26	Orthodontic vs. Orthognathic	-1.507	-2.902 to -0.1123	Yes	*	0.0307			
27	DTM vs. Orthognathic	-1.500	-2.895 to -0.1052	Yes	*	0.0317			
28									
29									
30	**Test details**	**Mean 1**	**Mean 2**	**Mean Diff.**	**SE of diff.**	**N1**	**N2**	**q**	**DF**

**2way ANOVA** Multiple comparisons									
31									
32	Mastication								
33	Orthodontic vs. DTM	0.9706	1.006	-0.03529	0.5905	17	17	0.08453	192.0
34	Orthodontic vs. Orthognathic	0.9706	2.635	-1.665	0.5905	17	17	3.987	192.0
35	DTM vs. Orthognathic	1.006	2.635	-1.629	0.5905	17	17	3.902	192.0
36									
37	Mobility								
38	Orthodontic vs. DTM	1.015	1.206	-0.1912	0.5905	17	17	0.4579	192.0
39	Orthodontic vs. Orthognathic	1.015	2.909	-1.894	0.5905	17	17	4.536	192.0
40	DTM vs. Orthognathic	1.206	2.909	-1.703	0.5905	17	17	4.078	192.0
41									
42	Communication								
43	Orthodontic vs. DTM	0.2353	0.05882	0.1765	0.5905	17	17	0.4226	192.0
44	Orthodontic vs. Orthognathic	0.2353	1.312	-1.076	0.5905	17	17	2.578	192.0
45	DTM vs. Orthognathic	0.05882	1.312	-1.253	0.5905	17	17	3.001	192.0
46									
47	Global								
48	Orthodontic vs. DTM	0.7224	0.7294	-0.007059	0.5905	17	17	0.01691	192.0
49	Orthodontic vs. Orthognathic	0.7224	2.229	-1.507	0.5905	17	17	3.609	192.0
50	DTM vs. Orthognathic	0.7294	2.229	-1.500	0.5905	17	17	3.592	192.0

Contingency		A	B	C
1	Table Analyzed	Frequency PHQ-9 DEPRESSION		
2				
3	**P value and statistical significance**			
4	Test	Chi-square		
5	Chi-square, df	106.0, 8		
6	P value	<0.0001		
7	P value summary	****		
8	One- or two-sided	NA		
9	Statistically significant (P < 0.05)?	Yes		
10				
11	**Data analyzed**			
12	Number of rows	5		
13	Number of columns	3		

	Linear reg.<br>Tabular results	A
1	**Best-fit values**	
2	Slope	0.005689
3	Y-intercept	-0.01281
4	X-intercept	2.252
5	1/slope	175.8
6		
7	**Std. Error**	
8	Slope	0.0001170
9	Y-intercept	0.01194
10		
11	**95% Confidence Intervals**	
12	Slope	0.005438 to 0.005940
13	Y-intercept	-0.03841 to 0.01279
14	X-intercept	-2.310 to 6.582
15		
16	**Goodness of Fit**	
17	R square	0.9941
18	Sy.x	0.03792
19		
20	**Is slope significantly non-zero?**	
21	F	2367
22	DFn, DFd	1, 14
23	P value	<0.0001
24	Deviation from zero?	Significant
25		
26	**Replicates test for lack of fit**	
27	SD replicates	0.03940
28	SD lack of fit	0.03586
29	Discrepancy (F)	0.8284
30	P value	0.5791

Linear reg. Tabular results		A
31	Evidence of inadequate model?	No
32		
33	**Equation**	Y = 0.005689*X - 0.01281
34		
35	**Data**	
36	Number of X values	16
37	Maximum number of Y replicates	2
38	Total number of values	16
39	Number of missing values	0

		X	A
		X (Interpolated)	Data Set-A (Entered)
		X	
1		332.696	1.880
2		46.721	0.253
3		124.411	0.695
4		44.261	0.239
5		22.817	0.117
6		124.938	0.698
7		140.582	0.787
8		27.211	0.142
9		122.478	0.684
10		569.808	3.229
11		110.525	0.616
12		99.803	0.555
13		243.582	1.373
14		164.135	0.921
15		92.773	0.515
16		46.721	0.253
17		238.309	1.343
18		47.424	0.257
19		74.317	0.410
20		43.733	0.236
21		92.245	0.512
22		34.769	0.185
23		61.310	0.336
24		53.928	0.294
25		188.742	1.061
26		55.158	0.301
27		221.611	1.248
28		4.361	0.012
29		58.849	0.322

		X	A
		X (Interpolated)	Data Set-A (Entered)
		X	
30		240.066	1.353
31		231.630	1.305
32		17.895	0.089
33		40.042	0.215
34		148.315	0.831
35		29.145	0.153
36		21.059	0.107
37		25.981	0.135
38		15.435	0.075
39		4.361	0.012
40		97.870	0.544
41		69.747	0.384
42		79.414	0.439
43		93.300	0.518
44		4.010	0.010
45		48.303	0.262
46		51.467	0.280
47		9.283	0.040
48		116.853	0.652
49		23.872	0.123
50		59.904	0.328
51		46.897	0.254
52		78.887	0.436
53		38.109	0.204
54		6.822	0.026

Linear reg. Tabular results		A
1	**Best-fit values**	
2	Slope	0.005689
3	Y-intercept	-0.01281
4	X-intercept	2.252
5	1/slope	175.8
6		
7	**Std. Error**	
8	Slope	0.0001170
9	Y-intercept	0.01194
10		
11	**95% Confidence Intervals**	
12	Slope	0.005438 to 0.005940
13	Y-intercept	-0.03841 to 0.01279
14	X-intercept	-2.310 to 6.582
15		
16	**Goodness of Fit**	
17	R square	0.9941
18	Sy.x	0.03792
19		
20	**Is slope significantly non-zero?**	
21	F	2367
22	DFn, DFd	1, 14
23	P value	<0.0001
24	Deviation from zero?	Significant
25		
26	**Replicates test for lack of fit**	
27	SD replicates	0.03940
28	SD lack of fit	0.03586
29	Discrepancy (F)	0.8284
30	P value	0.5791

**Linear reg.**<br>Tabular results		A
31	Evidence of inadequate model?	No
32		
33	**Equation**	Y = 0.005689*X - 0.01281
34		
35	**Data**	
36	Number of X values	16
37	Maximum number of Y replicates	2
38	Total number of values	16
39	Number of missing values	0

	X Seq.	A		
	X			
	X	Mean	+Error	-Error
1	**0.00**	-0.013	0.026	0.026
2	**1.25**	-0.006	0.025	0.025
3	**2.50**	0.001	0.025	0.025
4	**3.75**	0.009	0.025	0.025
5	**5.00**	0.016	0.025	0.025
6	**6.25**	0.023	0.025	0.025
7	**7.50**	0.030	0.025	0.025
8	**8.75**	0.037	0.024	0.024
9	**10.00**	0.044	0.024	0.024
10	**11.25**	0.051	0.024	0.024
11	**12.50**	0.058	0.024	0.024
12	**13.75**	0.065	0.024	0.024
13	**15.00**	0.073	0.024	0.024
14	**16.25**	0.080	0.023	0.023
15	**17.50**	0.087	0.023	0.023
16	**18.75**	0.094	0.023	0.023
17	**20.00**	0.101	0.023	0.023
18	**21.25**	0.108	0.023	0.023
19	**22.50**	0.115	0.023	0.023
20	**23.75**	0.122	0.022	0.022
21	**25.00**	0.129	0.022	0.022
22	**26.25**	0.137	0.022	0.022
23	**27.50**	0.144	0.022	0.022
24	**28.75**	0.151	0.022	0.022
25	**30.00**	0.158	0.022	0.022
26	**31.25**	0.165	0.022	0.022
27	**32.50**	0.172	0.022	0.022
28	**33.75**	0.179	0.022	0.022
29	**35.00**	0.186	0.021	0.021
30	**36.25**	0.193	0.021	0.021

	X Seq.	A		
	X			
	X	Mean	+Error	-Error
31	**37.50**	0.201	0.021	0.021
32	**38.75**	0.208	0.021	0.021
33	**40.00**	0.215	0.021	0.021
34	**41.25**	0.222	0.021	0.021
35	**42.50**	0.229	0.021	0.021
36	**43.75**	0.236	0.021	0.021
37	**45.00**	0.243	0.021	0.021
38	**46.25**	0.250	0.021	0.021
39	**47.50**	0.257	0.021	0.021
40	**48.75**	0.265	0.021	0.021
41	**50.00**	0.272	0.021	0.021
42	**51.25**	0.279	0.021	0.021
43	**52.50**	0.286	0.020	0.020
44	**53.75**	0.293	0.020	0.020
45	**55.00**	0.300	0.020	0.020
46	**56.25**	0.307	0.020	0.020
47	**57.50**	0.314	0.020	0.020
48	**58.75**	0.321	0.020	0.020
49	**60.00**	0.329	0.020	0.020
50	**61.25**	0.336	0.020	0.020
51	**62.50**	0.343	0.020	0.020
52	**63.75**	0.350	0.020	0.020
53	**65.00**	0.357	0.020	0.020
54	**66.25**	0.364	0.020	0.020
55	**67.50**	0.371	0.020	0.020
56	**68.75**	0.378	0.020	0.020
57	**70.00**	0.385	0.020	0.020
58	**71.25**	0.393	0.020	0.020
59	**72.50**	0.400	0.021	0.021
60	**73.75**	0.407	0.021	0.021

	X Seq.	A		
	X			
	X	Mean	+Error	-Error
61	**75.00**	0.414	0.021	0.021
62	**76.25**	0.421	0.021	0.021
63	**77.50**	0.428	0.021	0.021
64	**78.75**	0.435	0.021	0.021
65	**80.00**	0.442	0.021	0.021
66	**81.25**	0.449	0.021	0.021
67	**82.50**	0.457	0.021	0.021
68	**83.75**	0.464	0.021	0.021
69	**85.00**	0.471	0.021	0.021
70	**86.25**	0.478	0.021	0.021
71	**87.50**	0.485	0.021	0.021
72	**88.75**	0.492	0.021	0.021
73	**90.00**	0.499	0.022	0.022
74	**91.25**	0.506	0.022	0.022
75	**92.50**	0.513	0.022	0.022
76	**93.75**	0.521	0.022	0.022
77	**95.00**	0.528	0.022	0.022
78	**96.25**	0.535	0.022	0.022
79	**97.50**	0.542	0.022	0.022
80	**98.75**	0.549	0.022	0.022
81	**100.00**	0.556	0.022	0.022
82	**101.25**	0.563	0.023	0.023
83	**102.50**	0.570	0.023	0.023
84	**103.75**	0.577	0.023	0.023
85	**105.00**	0.585	0.023	0.023
86	**106.25**	0.592	0.023	0.023
87	**107.50**	0.599	0.023	0.023
88	**108.75**	0.606	0.023	0.023
89	**110.00**	0.613	0.024	0.024
90	**111.25**	0.620	0.024	0.024

	X Seq.	A		
	X			
	X	Mean	+Error	-Error
91	**112.50**	0.627	0.024	0.024
92	**113.75**	0.634	0.024	0.024
93	**115.00**	0.641	0.024	0.024
94	**116.25**	0.649	0.024	0.024
95	**117.50**	0.656	0.025	0.025
96	**118.75**	0.663	0.025	0.025
97	**120.00**	0.670	0.025	0.025
98	**121.25**	0.677	0.025	0.025
99	**122.50**	0.684	0.025	0.025
100	**123.75**	0.691	0.026	0.026
101	**125.00**	0.698	0.026	0.026
102	**126.25**	0.705	0.026	0.026
103	**127.50**	0.713	0.026	0.026
104	**128.75**	0.720	0.026	0.026
105	**130.00**	0.727	0.027	0.027
106	**131.25**	0.734	0.027	0.027
107	**132.50**	0.741	0.027	0.027
108	**133.75**	0.748	0.027	0.027
109	**135.00**	0.755	0.027	0.027
110	**136.25**	0.762	0.028	0.028
111	**137.50**	0.769	0.028	0.028
112	**138.75**	0.777	0.028	0.028
113	**140.00**	0.784	0.028	0.028
114	**141.25**	0.791	0.028	0.028
115	**142.50**	0.798	0.029	0.029
116	**143.75**	0.805	0.029	0.029
117	**145.00**	0.812	0.029	0.029
118	**146.25**	0.819	0.029	0.029
119	**147.50**	0.826	0.030	0.030
120	**148.75**	0.833	0.030	0.030

	X Seq.	A		
	X			
	X	Mean	+Error	-Error
121	**150.00**	0.841	0.030	0.030
122	**151.25**	0.848	0.030	0.030
123	**152.50**	0.855	0.030	0.030
124	**153.75**	0.862	0.031	0.031
125	**155.00**	0.869	0.031	0.031
126	**156.25**	0.876	0.031	0.031
127	**157.50**	0.883	0.031	0.031
128	**158.75**	0.890	0.032	0.032
129	**160.00**	0.897	0.032	0.032
130	**161.25**	0.905	0.032	0.032
131	**162.50**	0.912	0.032	0.032
132	**163.75**	0.919	0.033	0.033
133	**165.00**	0.926	0.033	0.033
134	**166.25**	0.933	0.033	0.033
135	**167.50**	0.940	0.033	0.033
136	**168.75**	0.947	0.034	0.034
137	**170.00**	0.954	0.034	0.034
138	**171.25**	0.961	0.034	0.034
139	**172.50**	0.969	0.034	0.034
140	**173.75**	0.976	0.035	0.035
141	**175.00**	0.983	0.035	0.035
142	**176.25**	0.990	0.035	0.035
143	**177.50**	0.997	0.035	0.035
144	**178.75**	1.004	0.036	0.036
145	**180.00**	1.011	0.036	0.036
146	**181.25**	1.018	0.036	0.036
147	**182.50**	1.025	0.036	0.036
148	**183.75**	1.033	0.037	0.037
149	**185.00**	1.040	0.037	0.037
150	**186.25**	1.047	0.037	0.037

	X Seq.	A		
	X			
	X	Mean	+Error	-Error
151	**187.50**	1.054	0.037	0.037
152	**188.75**	1.061	0.038	0.038
153	**190.00**	1.068	0.038	0.038
154	**191.25**	1.075	0.038	0.038
155	**192.50**	1.082	0.039	0.039
156	**193.75**	1.089	0.039	0.039
157	**195.00**	1.097	0.039	0.039
158	**196.25**	1.104	0.039	0.039
159	**197.50**	1.111	0.040	0.040
160	**198.75**	1.118	0.040	0.040
161	**200.00**	1.125	0.040	0.040
162	**201.25**	1.132	0.040	0.040
163	**202.50**	1.139	0.041	0.041
164	**203.75**	1.146	0.041	0.041
165	**205.00**	1.153	0.041	0.041
166	**206.25**	1.161	0.042	0.042
167	**207.50**	1.168	0.042	0.042
168	**208.75**	1.175	0.042	0.042
169	**210.00**	1.182	0.042	0.042
170	**211.25**	1.189	0.043	0.043
171	**212.50**	1.196	0.043	0.043
172	**213.75**	1.203	0.043	0.043
173	**215.00**	1.210	0.043	0.043
174	**216.25**	1.218	0.044	0.044
175	**217.50**	1.225	0.044	0.044
176	**218.75**	1.232	0.044	0.044
177	**220.00**	1.239	0.045	0.045
178	**221.25**	1.246	0.045	0.045
179	**222.50**	1.253	0.045	0.045
180	**223.75**	1.260	0.045	0.045

	X Seq.	A		
	X			
	X	Mean	+Error	-Error
181	**225.00**	1.267	0.046	0.046
182	**226.25**	1.274	0.046	0.046
183	**227.50**	1.282	0.046	0.046
184	**228.75**	1.289	0.047	0.047
185	**230.00**	1.296	0.047	0.047
186	**231.25**	1.303	0.047	0.047
187	**232.50**	1.310	0.047	0.047
188	**233.75**	1.317	0.048	0.048
189	**235.00**	1.324	0.048	0.048
190	**236.25**	1.331	0.048	0.048
191	**237.50**	1.338	0.048	0.048
192	**238.75**	1.346	0.049	0.049
193	**240.00**	1.353	0.049	0.049
194	**241.25**	1.360	0.049	0.049
195	**242.50**	1.367	0.050	0.050
196	**243.75**	1.374	0.050	0.050
197	**245.00**	1.381	0.050	0.050
198	**246.25**	1.388	0.050	0.050
199	**247.50**	1.395	0.051	0.051
200	**248.75**	1.402	0.051	0.051
201	**250.00**	1.410	0.051	0.051

**Linear reg.** Residual plot		X	A	
		X		
		X	A:1	A:2
1		0.000	0.033	0.030
2		3.906	0.009	0.008
3		7.825	-0.007	-0.005
4		15.625	-0.024	-0.016
5		31.250	-0.006	-0.017
6		62.500	-0.043	-0.018
7		125.000	0.067	0.005
8		250.000	-0.079	0.063

		X	A
		X (Interpolated)	Data Set-A (Entered)
		X	
1		332.696	1.880
2		46.721	0.253
3		124.411	0.695
4		44.261	0.239
5		22.817	0.117
6		124.938	0.698
7		140.582	0.787
8		27.211	0.142
9		122.478	0.684
10		569.808	3.229
11		110.525	0.616
12		99.803	0.555
13		243.582	1.373
14		164.135	0.921
15		92.773	0.515
16		46.721	0.253
17		238.309	1.343
18		47.424	0.257
19		74.317	0.410
20		43.733	0.236
21		92.245	0.512
22		34.769	0.185
23		61.310	0.336
24		53.928	0.294
25		188.742	1.061
26		55.158	0.301
27		221.611	1.248
28		4.361	0.012
29		58.849	0.322

		X	A
		X (Interpolated)	Data Set-A (Entered)
		X	
30		240.066	1.353
31		231.630	1.305
32		17.895	0.089
33		40.042	0.215
34		148.315	0.831
35		29.145	0.153
36		21.059	0.107
37		25.981	0.135
38		15.435	0.075
39		4.361	0.012
40		97.870	0.544
41		69.747	0.384
42		79.414	0.439
43		93.300	0.518
44		4.010	0.010
45		48.303	0.262
46		51.467	0.280
47		9.283	0.040
48		116.853	0.652
49		23.872	0.123
50		59.904	0.328
51		46.897	0.254
52		78.887	0.436
53		38.109	0.204
54		6.822	0.026

Ordinary one-way ANOVA<br>ANOVA results						
1	Table Analyzed	IL-1Beta				
2	Data sets analyzed	A-C				
3						
4	**ANOVA summary**					
5	F	4.871				
6	P value	0.0119				
7	P value summary	*				
8	Significant diff. among means (P < 0.05)?	Yes				
9	R square	0.1687				
10						
11	**Brown-Forsythe test**					
12	F (DFn, DFd)	2.424 (2, 48)				
13	P value	0.0993				
14	P value summary	ns				
15	Are SDs significantly different (P < 0.05)?	No				
16						
17	**Bartlett's test**					
18	Bartlett's statistic (corrected)	22.95				
19	P value	<0.0001				
20	P value summary	****				
21	Are SDs significantly different (P < 0.05)?	Yes				
22						
23	**ANOVA table**	**SS**	**DF**	**MS**	**F (DFn, DFd)**	**P value**
24	Treatment (between columns)	86900	2	43450	F (2, 48) = 4.8	P=0.0119
25	Residual (within columns)	428193	48	8921		
26	Total	515093	50			
27						
28	**Data summary**					
29	Number of treatments (columns)	3				
30	Number of values (total)	51				

Ordinary one-way ANOVA<br>Multiple comparisons									
1	Number of families	1							
2	Number of comparisons per family	3							
3	Alpha	0.05							
4									
5	**Tukey's multiple comparisons test**	**Mean Diff.**	**95.00% CI of diff.**	**Significant?**	**Summary**	**Adjusted P Value**			
6	Orthodontic vs. DTM	-40.03	-118.4 to 38.32	No	ns	0.4383	A-B		
7	Orthodontic vs. Orthognathic	-100.4	-178.8 to -22.08	Yes	**	0.0089	A-C		
8	DTM vs. Orthognathic	-60.39	-138.7 to 17.96	No	ns	0.1602	B-C		
9									
10	**Test details**	**Mean 1**	**Mean 2**	**Mean Diff.**	**SE of diff.**	**n1**	**n2**	**q**	**DF**
11	Orthodontic vs. DTM	49.68	89.71	-40.03	32.40	17	17	1.748	48
12	Orthodontic vs. Orthognathic	49.68	150.1	-100.4	32.40	17	17	4.384	48
13	DTM vs. Orthognathic	89.71	150.1	-60.39	32.40	17	17	2.636	48

Ordinary one-way ANOVA<br>ANOVA results						
1	Table Analyzed	IL-1Beta				
2	Data sets analyzed	A-C				
3						
4	**ANOVA summary**					
5	F	4.871				
6	P value	0.0119				
7	P value summary	*				
8	Significant diff. among means (P < 0.05)?	Yes				
9	R square	0.1687				
10						
11	**Brown-Forsythe test**					
12	F (DFn, DFd)	2.424 (2, 48)				
13	P value	0.0993				
14	P value summary	ns				
15	Are SDs significantly different (P < 0.05)?	No				
16						
17	**Bartlett's test**					
18	Bartlett's statistic (corrected)	22.95				
19	P value	<0.0001				
20	P value summary	****				
21	Are SDs significantly different (P < 0.05)?	Yes				
22						
23	**ANOVA table**	**SS**	**DF**	**MS**	**F (DFn, DFd)**	**P value**
24	Treatment (between columns)	86900	2	43450	F (2, 48) = 4.871	P=0.0119
25	Residual (within columns)	428193	48	8921		
26	Total	515093	50			
27						
28	**Data summary**					
29	Number of treatments (columns)	3				
30	Number of values (total)	51				

Ordinary one-way ANOVA<br>Multiple comparisons								
1	Number of families	1						
2	Number of comparisons per family	2						
3	Alpha	0.05						
4								
5	**Dunnett's multiple comparisons test**	**Mean Diff.**	**95.00% CI of diff.**	**Significant?**	**Summary**	**Adjusted P Value**	**A-?**	
6	Orthodontic vs. DTM	-40.03	-113.9 to 33.80	No	ns	0.3660	B	DTM
7	Orthodontic vs. Orthognathic	-100.4	-174.3 to -26.59	Yes	**	0.0062	C	Orthognathic
8								
9	**Test details**	**Mean 1**	**Mean 2**	**Mean Diff.**	**SE of diff.**	**n1**	**n2**	**q**
10	Orthodontic vs. DTM	49.68	89.71	-40.03	32.40	17	17	1.236
11	Orthodontic vs. Orthognathic	49.68	150.1	-100.4	32.40	17	17	3.100

1	
2	
3	
4	
5	
6	
7	
8	
9	**DF**
10	48
11	48

Contingency		A	B	C
1	Table Analyzed	Frequency CPG		
2				
3	**P value and statistical significance**			
4	Test	Chi-square		
5	Chi-square, df	126.4, 8		
6	P value	<0.0001		
7	P value summary	****		
8	One- or two-sided	NA		
9	Statistically significant (P < 0.05)?	Yes		
10				
11	**Data analyzed**			
12	Number of rows	5		
13	Number of columns	3		

Contingency		A	B	C
1	Table Analyzed	DC		
2				
3	**P value and statistical significance**			
4	Test	Chi-square		
5	Chi-square, df	12.59, 4		
6	P value	0.0134		
7	P value summary	*		
8	One- or two-sided	NA		
9	Statistically significant (P < 0.05)?	Yes		
10				
11	**Data analyzed**			
12	Number of rows	3		
13	Number of columns	3		

	Unpaired t test	
1	Table Analyzed	ORTHO VS DTM
2		
3	Column B	DTM
4	vs.	vs.
5	Column A	ORTHODONTIC
6		
7	**Unpaired t test**	
8	P value	0.0749
9	P value summary	ns
10	Significantly different (P < 0.05)?	No
11	One- or two-tailed P value?	Two-tailed
12	t, df	t=1.841, df=32
13		
14	**How big is the difference?**	
15	Mean of column A	49.68
16	Mean of column B	89.71
17	Difference between means (B - A) ± SEM	40.03 ± 21.74
18	95% confidence interval	-4.252 to 84.32
19	R squared (eta squared)	0.09580
20		
21	**F test to compare variances**	
22	F, DFn, Dfd	5.249, 16, 16
23	P value	0.0019
24	P value summary	**
25	Significantly different (P < 0.05)?	Yes
26		
27	**Data analyzed**	
28	Sample size, column A	17
29	Sample size, column B	17

**Ordinary one-way ANOVA**<br>ANOVA results						
1	Table Analyzed	Glutamate				
2	Data sets analyzed	A-C				
3						
4	**ANOVA summary**					
5	F	1.166				
6	P value	0.3219				
7	P value summary	ns				
8	Significant diff. among means (P < 0.05)?	No				
9	R square	0.05510				
10						
11	**Brown-Forsythe test**					
12	F (DFn, DFd)	0.7005 (2, 40)				
13	P value	0.5023				
14	P value summary	ns				
15	Are SDs significantly different (P < 0.05)?	No				
16						
17	**Bartlett's test**					
18	Bartlett's statistic (corrected)	2.183				
19	P value	0.3357				
20	P value summary	ns				
21	Are SDs significantly different (P < 0.05)?	No				
22						
23	**ANOVA table**	**SS**	**DF**	**MS**	**F (DFn, DFd)**	**P value**
24	Treatment (between columns)	698882	2	349441	F (2, 40) = 1.166	P=0.3219
25	Residual (within columns)	11984982	40	299625		
26	Total	12683864	42			
27						
28	**Data summary**					
29	Number of treatments (columns)	3				
30	Number of values (total)	43				

Ordinary one-way ANOVA
Multiple comparisons

1	Number of families	1							
2	Number of comparisons per family	3							
3	Alpha	0.05							
4									
5	**Tukey's multiple comparisons test**	**Mean Diff.**	**95.00% CI of diff.**	**Significant?**	**Summary**	**Adjusted P Value**			
6	Orthodontic vs. DTM	266.5	-256.1 to 789.1	No	ns	0.4363	A-B		
7	Orthodontic vs. Orthognathic	285.2	-205.7 to 776.0	No	ns	0.3435	A-C		
8	DTM vs. Orthognathic	18.66	-472.2 to 509.5	No	ns	0.9953	B-C		
9									
10	**Test details**	**Mean 1**	**Mean 2**	**Mean Diff.**	**SE of diff.**	**n1**	**n2**	**q**	**DF**
11	Orthodontic vs. DTM	1103	836.1	266.5	214.7	13	13	1.755	40
12	Orthodontic vs. Orthognathic	1103	817.4	285.2	201.7	13	17	2.000	40
13	DTM vs. Orthognathic	836.1	817.4	18.66	201.7	13	17	0.1308	40

Descriptive statistics		A	B	C
		Orthodontic	DTM	Orthognathic
1	Number of values	13	13	17
2				
3	Minimum	313.2	0.000	0.000
4	Maximum	1746	2054	2007
5	Range	1433	2054	2007
6				
7	Mean	1103	836.1	817.4
8	Std. Deviation	405.4	593.3	601.5
9	Std. Error of Mean	112.4	164.6	145.9

**Identify outliers** Cleaned data		A	B	C
		Orthodontic	DTM	Orthognathic
1		1745.700	0.000	2007.200
2		971.100	773.300	0.000
3		1602.500	2054.100	0.000
4		1013.400	378.600	1709.400
5		1318.800	1936.500	567.500
6		1372.900	836.600	570.700
7		1386.500	834.600	769.700
8		562.600	861.500	
9		313.200	1095.300	1657.000
10		1154.400	482.500	1221.800
11		964.400	555.000	917.100
12		731.900	812.000	932.700
13		1196.100	248.900	300.900
14				660.500
15				495.500
16				411.800
17				1413.300
18				260.900

**Identify outliers**<br>Outliers		A	B	C
		Orthodontic	DTM	Orthognathic

Identify outliers<br>Summary		A<br>Orthodontic	B<br>DTM	C<br>Orthognathic
1	**Method**			
2	ROUT (Q = 1%)			
3				
4	**Number of points**			
5	# Y values analyzed	13	13	17
6	Outliers	0	0	0

Transform		A	B	C
		Orthodontic	DTM	Orthognathic
1		175.000	725.000	94.000
2		311.000	468.000	252.000
3		75.000	206.000	601.000
4		42.000	132.000	261.000
5		63.000	77.000	95.000
6		127.000	22.000	352.000
7		0.000	0.000	54.000
8		0.000	9.000	
9		4.000	0.000	82.000
10		0.000	5.000	27.000
11		155.000	0.000	0.000
12		156.000	0.000	0.000
13		0.000	977.000	73.000
14				0.000
15				0.000
16				0.000
17				0.000
18				0.000

Descriptive statistics		A	B	C
		Orthodontic	DTM	Orthognathic
1	Number of values	13	13	17
2				
3	Minimum	0.000	0.000	0.000
4	Maximum	311.0	977.0	601.0
5	Range	311.0	977.0	601.0
6				
7	Mean	85.23	201.6	111.2
8	Std. Deviation	95.21	321.1	165.8
9	Std. Error of Mean	26.41	89.05	40.22

Ordinary one-way ANOVA<br>ANOVA results						
1	Table Analyzed	Transform of Serotonin				
2	Data sets analyzed	A-C				
3						
4	**ANOVA summary**					
5	F	1.105				
6	P value	0.3410				
7	P value summary	ns				
8	Significant diff. among means (P < 0.05)?	No				
9	R square	0.05237				
10						
11	**Brown-Forsythe test**					
12	F (DFn, DFd)	1.469 (2, 40)				
13	P value	0.2422				
14	P value summary	ns				
15	Are SDs significantly different (P < 0.05)?	No				
16						
17	**Bartlett's test**					
18	Bartlett's statistic (corrected)	16.29				
19	P value	0.0003				
20	P value summary	***				
21	Are SDs significantly different (P < 0.05)?	Yes				
22						
23	**ANOVA table**	**SS**	**DF**	**MS**	**F (DFn, DFd)**	**P value**
24	Treatment (between columns)	98695	2	49347	F (2, 40) = 1.105	P=0.3410
25	Residual (within columns)	1785840	40	44646		
26	Total	1884535	42			
27						
28	**Data summary**					
29	Number of treatments (columns)	3				
30	Number of values (total)	43				

Ordinary one-way ANOVA<br>Multiple comparisons									
1	Number of families	1							
2	Number of comparisons per family	3							
3	Alpha	0.05							
4									
5	**Holm-Sidak's multiple comparisons test**	**Mean Diff.**	**Significant?**	**Summary**	**Adjusted P Value**				
6	Orthodontic vs. DTM	-116.4	No	ns	0.4240	A-B			
7	Orthodontic vs. Orthognathic	-26.00	No	ns	0.7401	A-C			
8	DTM vs. Orthognathic	90.38	No	ns	0.4413	B-C			
9									
10	**Test details**	**Mean 1**	**Mean 2**	**Mean Diff.**	**SE of diff.**	**n1**	**n2**	**t**	**DF**
11	Orthodontic vs. DTM	85.23	201.6	-116.4	82.88	13	13	1.404	40
12	Orthodontic vs. Orthognathic	85.23	111.2	-26.00	77.85	13	17	0.3340	40
13	DTM vs. Orthognathic	201.6	111.2	90.38	77.85	13	17	1.161	40

Contingency		A	B	C
1	Table Analyzed	Educational Level		
2				
3	**P value and statistical significance**			
4	Test	Chi-square		
5	Chi-square, df	11.08, 4		
6	P value	0.0257		
7	P value summary	*		
8	One- or two-sided	NA		
9	Statistically significant (P < 0.05)?	Yes		
10				
11	**Data analyzed**			
12	Number of rows	3		
13	Number of columns	3		

Contingency		A	B	C
1	Table Analyzed	Parafunctional Habits		
2				
3	**P value and statistical sigr**			
4	Test	Chi-square		
5	Chi-square, df	15.97, 2		
6	P value	0.0003		
7	P value summary	***		
8	One- or two-sided	NA		
9	Statistically significant (P < 0	Yes		
10				
11	**Data analyzed**			
12	Number of rows	3		
13	Number of columns	2		

Contingency		A	B	C
1	Table Analyzed	Educational Level		
2				
3	**P value and statistical significance**			
4	Test	Chi-square		
5	Chi-square, df	11.08, 4		
6	P value	0.0257		
7	P value summary	*		
8	One- or two-sided	NA		
9	Statistically significant (P < 0.05)?	Yes		
10				
11	**Data analyzed**			
12	Number of rows	3		
13	Number of columns	3		

Contingency		A	B	C
1	Table Analyzed	Ethnicity		
2				
3	**P value and statistical significance**			
4	Test	Chi-square		
5	Chi-square, df	2.040, 2		
6	P value	0.3606		
7	P value summary	ns		
8	One- or two-sided	NA		
9	Statistically significant (P < 0.05)?	No		
10				
11	**Data analyzed**			
12	Number of rows	3		
13	Number of columns	2		

Contingency		A	B	C
1	Table Analyzed	Angle Class		
2				
3	**P value and statistical significance**			
4	Test	Chi-square		
5	Chi-square, df	51.00, 4		
6	P value	<0.0001		
7	P value summary	****		
8	One- or two-sided	NA		
9	Statistically significant (P < 0.05)?	Yes		
10				
11	**Data analyzed**			
12	Number of rows	3		
13	Number of columns	3		

Contingency		A	B	C
1	Table Analyzed	Complaint		
2				
3	**P value and statistical significance**			
4	Test	Chi-square		
5	Chi-square, df	35.55, 4		
6	P value	<0.0001		
7	P value summary	****		
8	One- or two-sided	NA		
9	Statistically significant (P < 0.05)?	Yes		
10				
11	**Data analyzed**			
12	Number of rows	3		
13	Number of columns	3		

Contingency		A	B	C
1	Table Analyzed	DC		
2				
3	**P value and statistical significance**			
4	Test	Chi-square		
5	Chi-square, df	12.59, 4		
6	P value	0.0134		
7	P value summary	*		
8	One- or two-sided	NA		
9	Statistically significant (P < 0.05)?	Yes		
10				
11	**Data analyzed**			
12	Number of rows	3		
13	Number of columns	3		

Correlation		A	B	C	D	E	F	G
		IL-1beta vs. CPI	IL-1beta vs. CPG	IL-1beta vs. OBC	IL-1beta vs. PHQ-9	IL-1beta vs. GAD-7	IL-1beta vs. PHQ-15	IL-1beta vs. OHIP
1	**Pearson r**							
2	r	-0.2417	-0.1644	-0.1954	-0.1659	0.01627	-0.1022	-0.1641
3	95% confidence interval	-0.6472 to 0.2703	-0.5978 to 0.3434	-0.6180 to 0.3148	-0.5988 to 0.3420	-0.4680 to 0.4931	-0.5555 to 0.3980	-0.5976 to 0.3437
4	R squared	0.05842	0.02703	0.03818	0.02751	0.0002647	0.01044	0.02692
5								
6	**P value**							
7	P (two-tailed)	0.3500	0.5283	0.4523	0.5246	0.9506	0.6964	0.5292
8	P value summary	ns	ns	ns	ns	ns	ns	ns
9	Significant? (alpha = 0.05)	No	No	No	No	No	No	No
10								
11	**Number of XY Pairs**	17	17	17	17	17	17	17

	Correlation	A	B	C	D	E	F	G
		Glutamate vs. CPI	Glutamate vs. CPG	Glutamate vs. OBC	Glutamate vs. PHQ-9	Glutamate vs. GAD-7	Glutamate vs. PHQ-15	Glutamate vs. OHIP
1	**Pearson r**							
2	r	0.3230	0.6025	0.4917	0.2147	0.4967	0.4239	0.6284
3	95% confidence interval	-0.1866 to 0.6956	0.1715 to 0.8399	0.01445 to 0.7865	-0.2966 to 0.6303	0.02112 to 0.7890	-0.07127 to 0.7514	0.2117 to 0.8518
4	R squared	0.1043	0.3630	0.2417	0.04609	0.2467	0.1797	0.3949
5								
6	**P value**							
7	P (two-tailed)	0.2061	0.0105	0.0450	0.4080	0.0425	0.0899	0.0069
8	P value summary	ns	*	*	ns	*	ns	**
9	Significant? (alpha = 0.05)	No	Yes	Yes	No	Yes	No	Yes
10								
11	**Number of XY Pairs**	17	17	17	17	17	17	17

	Correlation	A	B	C	D	E	F	G
		Serotonin vs. CPI	Serotonin vs. CPG	Serotonin vs. OBC	Serotonin vs. PHQ-9	Serotonin vs. GAD-7	Serotonin vs. PHQ-15	Serotonin vs. OHIP
1	**Pearson r**							
2	r	0.4766	0.1547	0.2729	-0.2771	-0.2271	-0.2387	-0.1226
3	95% confidence interval	-0.005251 to 0.7788	-0.3521 to 0.5914	-0.2391 to 0.6662	-0.6687 to 0.2348	-0.6381 to 0.2846	-0.6453 to 0.2733	-0.5697 to 0.3804
4	R squared	0.2271	0.02394	0.07447	0.07680	0.05158	0.05697	0.01504
5								
6	**P value**							
7	P (two-tailed)	0.0531	0.5532	0.2893	0.2815	0.3807	0.3562	0.6391
8	P value summary	ns	ns	ns	ns	ns	ns	ns
9	Significant? (alpha = 0.05)	No	No	No	No	No	No	No
10								
11	**Number of XY Pairs**	17	17	17	17	17	17	17

Descriptive statistics		A	B	C
		Orthodontic	DTM	Orthognathic
1	Number of values	17	17	17
2				
3	Minimum	19.00	18.00	16.00
4	Maximum	47.00	66.00	51.00
5	Range	28.00	48.00	35.00
6				
7	Mean	27.18	36.53	21.94
8	Std. Deviation	8.025	16.25	7.838
9	Std. Error of Mean	1.946	3.942	1.901

**Ordinary one-way ANOVA**<br>ANOVA results						
1	Table Analyzed	Age mean				
2	Data sets analyzed	A-C				
3						
4	**ANOVA summary**					
5	F	7.143				
6	P value	0.0019				
7	P value summary	**				
8	Significant diff. among means (P < 0.05)?	Yes				
9	R square	0.2294				
10						
11	**Brown-Forsythe test**					
12	F (DFn, DFd)	6.396 (2, 48)				
13	P value	0.0034				
14	P value summary	**				
15	Are SDs significantly different (P < 0.05)?	Yes				
16						
17	**Bartlett's test**					
18	Bartlett's statistic (corrected)	11.56				
19	P value	0.0031				
20	P value summary	**				
21	Are SDs significantly different (P < 0.05)?	Yes				
22						
23	**ANOVA table**	**SS**	**DF**	**MS**	**F (DFn, DFd)**	**P value**
24	Treatment (between columns)	1857	2	928.5	F (2, 48) = 7.143	P=0.0019
25	Residual (within columns)	6240	48	130.0		
26	Total	8097	50			
27						
28	**Data summary**					
29	Number of treatments (columns)	3				
30	Number of values (total)	51				

Ordinary one-way ANOVA
Multiple comparisons

1	Number of families	1							
2	Number of comparisons per family	3							
3	Alpha	0.05							
4									
5	**Tukey's multiple comparisons test**	**Mean Diff.**	**95.00% CI of diff.**	**Significant?**	**Summary**	**Adjusted P Value**			
6	Orthodontic vs. DTM	-9.353	-18.81 to 0.1049	No	ns	0.0532	A-B		
7	Orthodontic vs. Orthognathic	5.235	-4.223 to 14.69	No	ns	0.3811	A-C		
8	DTM vs. Orthognathic	14.59	5.130 to 24.05	Yes	**	0.0014	B-C		
9									
10	**Test details**	**Mean 1**	**Mean 2**	**Mean Diff.**	**SE of diff.**	**n1**	**n2**	**q**	**DF**
11	Orthodontic vs. DTM	27.18	36.53	-9.353	3.911	17	17	3.382	48
12	Orthodontic vs. Orthognathic	27.18	21.94	5.235	3.911	17	17	1.893	48
13	DTM vs. Orthognathic	36.53	21.94	14.59	3.911	17	17	5.276	48

Contingency		A	B	C
1	Table Analyzed	Frequency GAD-7 ANXIETY		
2				
3	**P value and statistical significance**			
4	Test	Chi-square		
5	Chi-square, df	125.7, 6		
6	P value	<0.0001		
7	P value summary	****		
8	One- or two-sided	NA		
9	Statistically significant (P < 0.05)?	Yes		
10				
11	**Data analyzed**			
12	Number of rows	4		
13	Number of columns	3		

Contingency		A	B	C
1	Table Analyzed	Frequency PHQ-15 PHYSICAL SYMPTOMS		
2				
3	**P value and statistical significance**			
4	Test	Chi-square		
5	Chi-square, df	97.93, 6		
6	P value	<0.0001		
7	P value summary	****		
8	One- or two-sided	NA		
9	Statistically significant (P < 0.05)?	Yes		
10				
11	**Data analyzed**			
12	Number of rows	4		
13	Number of columns	3		

Contingency		A	B	C
1	Table Analyzed	Frequency OBC		
2				
3	**P value and statistical significance**			
4	Test	Chi-square		
5	Chi-square, df			
6	P value			
7	P value summary			
8	One- or two-sided			
9	Statistically significant (P < 0.05)?			
10				
11	**Data analyzed**			
12	Number of rows	3		
13	Number of columns	3		

Contingency		A	B	C
1	Table Analyzed	Sex		
2				
3	**P value and statistical significance**			
4	Test	Chi-square		
5	Chi-square, df	0.7286, 2		
6	P value	0.6947		
7	P value summary	ns		
8	One- or two-sided	NA		
9	Statistically significant (P < 0.05)?	No		
10				
11	**Data analyzed**			
12	Number of rows	3		
13	Number of columns	2		

Contingency		A	B	C
1	Table Analyzed	Copy of DC		
2				
3	**P value and statistical significance**			
4	Test	Chi-square		
5	Chi-square, df	2.923, 2		
6	P value	0.2318		
7	P value summary	ns		
8	One- or two-sided	NA		
9	Statistically significant (P < 0.05)?	No		
10				
11	**Data analyzed**			
12	Number of rows	2		
13	Number of columns	3		

Descriptive statistics		A	B
1	Number of values	17	17
2			
3	Minimum	4.010	4.361
4	Maximum	116.9	240.1
5	Range	112.8	235.7
6			
7	Mean	49.68	89.71
8	Std. Deviation	35.86	82.16
9	Std. Error of Mean	8.697	19.93
