# Supplementary material for: Assessment of temporomandibular disorders and their relationship with life quality and salivary biomarkers in patients with dentofacial deformities: A clinical observational study
Source: PLoS One. 2023 Jul 20;18(7):e0288914. doi: 10.1371/journal.pone.0288914 (PMC10358945; doi:10.1371/journal.pone.0288914)
Supplement: Attachment — Submitted filename: Response to Reviewers.pdf [file pone.0288914.s003.pdf]

Manuscript number PONE-D-23-12302
Response to Editor

1. Please ensure that your manuscript meets PLOS ONE's style requirements, including those for file naming.
The PLOS ONE style templates can be found at
https://journals.plos.org/plosone/s/file?id=wjVg/PLOSOne_formatting_sample_main_body.pdf
and
https://journals.plos.org/plosone/s/file?id=ba62/PLOSOne_formatting_sample_title_authors_affiliations.pdf
As recommended, we have thoroughly revised the style requirements and the file names in the revised version of our manuscript. We hope that the new version of the paper is now in total accord with the Journal formatting requirements.

2. Please note that funding information should not appear in any section or other areas of your manuscript. We will only publish funding information present in the Funding Statement section of the online submission form. Please remove any funding-related text from the manuscript.
"This work was supported by grants from Coordenação de Aperfeiçoamento de Nível Superior (CAPES; Financial Code 001); Conselho Nacional de Desenvolvimento Científico e Tecnológico (CNPq); and Fundação de Amparo à Pesquisa do Estado do Rio Grande do Sul (FAPERGS). B.B.C. is a MSc. Student receiving grants from CAPES. M.M.C. is a research career awardee of CNPq (304042/2018-8)"
We apologize for this imprecision. Please, observe that we have removed the funding information from the revised version of the manuscript.

3. Thank you for stating the following financial disclosure:
"BBC - (001) Coordenação de Aperfeiçoamento de Pessoal de Nível Superior
NVB - (001) Coordenação de Aperfeiçoamento de Pessoal de Nível Superior
MMC - (304042/2018-8) Conselho nacional de Desenvolvimento Científico e Tecnológico "
Please state what role the funders took in the study. If the funders had no role, please state: "The funders had no role in study design, data collection and analysis, decision to publish, or preparation of the manuscript."
If this statement is not correct you must amend it as needed.
Please include this amended Role of Funder statement in your cover letter; we will change the online submission form on your behalf.
Thank you very much. As recommended, we have included a disclosure statement indicating that Funders had no role in the study, within the cover letter to the revised version of the paper.

4. We note that you have indicated that data from this study are available upon request. PLOS only allows data to be available upon request if there are legal or ethical restrictions on sharing data publicly. For more information on unacceptable data access restrictions, please see http://journals.plos.org/plosone/s/data-availability#loc-unacceptable-data-access-restrictions.
In your revised cover letter, please address the following prompts:
a) If there are ethical or legal restrictions on sharing a de-identified data set, please explain them in detail (e.g., data contain potentially sensitive information, data are owned by a third-party organization, etc.) and who has imposed them (e.g., an ethics committee). Please also provide contact information for a data access committee, ethics committee, or other institutional body to which data requests may be sent.
b) If there are no restrictions, please upload the minimal anonymized data set necessary to replicate your study findings as either Supporting Information files or to a stable, public repository and provide us with the relevant URLs, DOIs, or accession numbers. For a list of acceptable repositories, please see http://journals.plos.org/plosone/s/data-availability#loc-recommended-repositories.

We will update your Data Availability statement on your behalf to reflect the information you provide.
We apologize for this imprecision. Please, note that we have now uploaded the raw data as a PDF supplementary file, accompanying the revised version of the paper. This information has also been added in the cover letter to the revised manuscript.

5. We note that you have stated that you will provide repository information for your data at acceptance. Should your manuscript be accepted for publication, we will hold it until you provide the relevant accession numbers or DOIs necessary to access your data. If you wish to make changes to your Data Availability statement, please describe these changes in your cover letter and we will update your Data Availability statement to reflect the information you provide.
As mentioned in the item 4, we have included the raw data regarding the results of our manuscript as a supplementary file, when submitting the revised version of our manuscript.

6. Please include captions for your Supporting Information files at the end of your manuscript, and update any in-text citations to match accordingly. Please see our Supporting Information guidelines for more information: http://journals.plos.org/plosone/s/supporting-information.
Thank you very much for this comment. As required, we have corrected the text regarding the Supporting Information file of our manuscript.

Additional Editor comments:
Please modify the paper according to the reviewers' suggestions. The paper will be reconsidered for publication.
Thank you very much for the careful revision of our manuscript. Please, note that we have revised the paper according to the comments of both Reviewers. We hope that our manuscript has been satisfactorily improved to allow its acceptance in the present form.

Manuscript number PONE-D-23-12302
Response to Reviewer 1

Reviewer #1:

Dear Authors,
thank you for giving me the opportunity to revise your paper entitled " Screening for temporomandibular disorders in patients with dentofacial deformities: impacts on life quality and salivary biomarkers". The paper aims to explore the incidence of TMD in dentofacial deformities. The MS is well written and succinct, but there are some critical issues to address that makes the paper unsuitable for publication in Journal.
Thank you very much for evaluating our paper. We have responded to your comments below and the paper has been revised accordingly. The corrections will certainly help to improve the scientific level of our manuscript. We hope that the revised version of the paper has been satisfactorily improved to permit the paper to be published in PLOS ONE.

1. The Title is quite confuse and doesn't explain the study sample. Please, change it
Thank very much for this comment. Please, observe that we have re-written the Title in order to address your concerns, also considering the comments of Reviewer 2. Thus, the paper is now entitled "Assessment of temporomandibular disorders and their relationship with life quality and salivary biomarkers in patients with dentofacial deformities: a clinical observational study".

2. Please, delete in Abstract the sentence "in orthodontic treatment before surgical correction; n=17 each"
Thank you very much for this comment. As recommended, we have removed the text in parenthesis.

3. The introduction is quite poor of different clinical information. I suggest to improve it in terms of possible therapeutic approaches in TMD. Please, refer also to new conservative approaches ("Marotta N, Ferrillo M, Demeco A, Drago Ferrante V, Inzitari MT, Pellegrino R, Pino I, Russo I, de Sire A, Ammendolia A. Effects of Radial Extracorporeal Shock Wave Therapy in Reducing Pain in Patients with Temporomandibular Disorders: A Pilot Randomized Controlled Trial. Applied Sciences. 2022"; 12(8):3821. https://doi.org/10.3390/app12083821)
Thank you very much for this valuable criticism. Please, note that we have made an effort to improve the introduction section regarding the possible therapeutic approaches for TMD, also including the paper indicated by this Reviewer, in addition to another reference (Brighenti N, Battaglino A, Sinatti P, Abuín-Porras V, Sánchez Romero EA, Pedersini P, Villafañe JH. Effects of an Interdisciplinary Approach in the Management of Temporomandibular Disorders: A Scoping Review. Int J Environ Res Public Health. 2023 Feb 4;20(4):2777. doi: 10.3390/ijerph20042777). We hope this is now suitable to allow the publication of the manuscript in the present form.

4. Please, add the study design in the introduction
As recommended, we have added the study design in the introduction section, in the last paragraph. Thank you for this criticism

5. Please, delete in Material and Methods the sentence in brackets (n=17 each).
As suggested, we have removed "(n-17 each)".

6. Results are well written
Thank you very much for this positive comment.

7. Please, add the clinical implication of your founding
As indicated, we have included a statement with the study clinical implications at the end of the conclusion.

8. Please, add the Conclusion
As recommended, we have separated the last paragraph of the Discussion as the item "Conclusion"

Best Regards

Thank you very much for all the valuable comments regarding our manuscript. Your appointments certainly contributed to improve the quality of our study.

Manuscript number PONE-D-23-12302
Response to reviewer 2

Reviewer #2:

Dear Corresponding Author,

The paper is really interesting, well conducted and fits the objectives of the journal; but it is necessary to review some points in order to improve the quality of the paper.
Thank you very much for revising our manuscript. Your comments will certainly contribute to improve the paper's quality and readability.

1) First, i ask you to check the plagiarism of your article using Ithenticate.
Thank you very much for your recommendation. Accordingly, we have checked the manuscript plagiarism by using the tool Turnitin, which is freely available in our Institution. Please, note that we have included the file with similarity analysis showing that parts of text that had been marked are mostly related to methodological aspects (such as questionnaire descriptions) or specific terms and author's affiliation, without any characterization of plagiarism. The details regarding the maximal percentages of similarity are at the end of the PDF file.

2) About the Title of the article, I suggest you to modify it and add the type of article.
Thank very much for this comment. Please, observe that we have re-written the Title in order to address your concerns, also considering the comments of Reviewer 1. Thus, the paper is now entitled "Assessment of temporomandibular disorders and their relationship with life quality and salivary biomarkers in patients with dentofacial deformities: a clinical observational study".

3) The Abstract is precisely written, and the aim of the study is mentioned.
Thank you very much for the positive comments about our abstract. Thank you again for the careful revision of our paper.

4) The introduction section is very short and is needed to add other references to increase the quality of the manuscript. I suggest you add some lines on the impact of quality of life in patients with TMD and the prevalence of TMD in children and adolescents. I suggest including the following articles in the bibliography to enhance the impact of the paper. [10.1111/joor.13446]; [10.1111/joor.13472]
Thank you very much for your comments. Please, observe that we have extended the Introduction section of the study, by adding the abovementioned references, in addition to other two new references.

5) The Material and Methods section is adequate and well organized, but punctuation and spaces between words should be reviewed.
Thank you very much for this comment and the positive feedback. As recommended this sections has been thoroughly revised regarding the spaces and punctuation.

6) The conclusion is in accordance with the objectives of the research, its results and their interpretation, as well as the relevant literature. I suggest you include a section on study limitation.
Thank you very much. As recommended, we have included a separated paragraph with the main study limitations.

Regards
We would like to thank this Reviewer again for the suggestions and criticisms regarding our manuscript.
